# Resting state fast brain dynamics predict interindividual variability in motor performance

**DOI:** 10.1038/s41598-022-08767-z

**Published:** 2022-03-29

**Authors:** Liliia Roshchupkina, Vincent Wens, Nicolas Coquelet, Xavier de Tiege, Philippe Peigneux

**Affiliations:** 1 UR2NF—Neuropsychology and Functional Neuroimaging Research Unit affiliated at CRCN - Centre for Research in Cognition and Neurosciences, Avenue F.D. Roosevelt 50, 1050 Bruxelles, Belgium; 2 UNI—ULB Neuroscience Institute, Université Libre de Bruxelles (ULB), Avenue F.D. Roosevelt 50, 1050 Bruxelles, Belgium; 3 Laboratoire de Cartographie Fonctionnelle du Cerveau (LCFC), Université Libre de Bruxelles (ULB), Brussels, Belgium; 4 Department of Functional Neuroimaging, Service of Nuclear Medicine, CUB Hôpital Erasme, Université Libre de Bruxelles (ULB), Brussels, Belgium

**Keywords:** Neuroscience, Psychology

## Abstract

Motor learning features rapid enhancement during practice then offline post-practice gains with the reorganization of related brain networks. We hypothesised that fast transient, sub-second variations in magnetoencephalographic (MEG) network activity during the resting-state (RS) reflect early learning-related plasticity mechanisms and/or interindividual motor variability in performance. MEG RS activity was recorded before and 20 min after motor learning. Hidden Markov modelling (HMM) of MEG power envelope signals highlighted 8 recurrent topographical states. For two states, motor performance levels were associated with HMM temporal parameters both in pre- and post-learning resting-state sessions. However, no association emerged with offline changes in performance. These results suggest a trait-like relationship between spontaneous transient neural dynamics at rest and interindividual variations in motor abilities. On the other hand, transient RS dynamics seem not to be state-dependent, i.e., modulated by learning experience and reflect neural plasticity, at least on the short timescale.

## Introduction

Motor learning (ML) is a dynamic process subtending efficient daily life functioning. Typically, ML requires many repetitions to become effective in facilitating swift and accurate movement execution. Rapid changes in performance are observed during motor task practice (i.e., online improvement), whereas slower gains take place outside of practice (i.e., offline) during post-training periods^1,2^. Dynamic performance changes over time suggest that ML undergoes critical periods within this fast-slow framework. Indeed, the offline evolution of motor performance is characterised by a spontaneous improvement 30 min after practice (i.e., boost period), which is no longer observed when tested a few hours later (i.e., silent period)^3,4^, at which point performance remains at end-of-learning levels. A further performance increment takes place overnight. Performance levels achieved at the early, short-lived boost phase were found predictive of offline performance improvement 48 h later^4^, suggesting functional relevance of immediate post-training periods for the rapid reorganization of neural networks supporting ML and its consolidation in the long term^1–4^.

Neuroimaging studies provided insights into the neuroanatomical underpinnings of ML, and their functional interactions in relevant brain networks. Nowadays, functional connectivity measures allow investigating the functional brain network architecture both during actual ML practice (i.e., task-based connectivity) and “inactive” periods before and after learning (i.e., resting-state [RS] connectivity). Brain network features during task practice such as flexibility^5^, local path length, connectivity strength, and nodal efficiency^6^ not only change as a result of ML but are also predictive of future learning levels^5^. As well, functional network connectivity derived from RS measurements before a motor task predicts individual ML abilities^7^, whereas post-learning RS features were proposed to reflect task-induced plasticity^6,8^. Many studies investigated in detail the brain’s spatial components and their interplay in ML, but its underlying temporal neural dynamics are still poorly understood.

Temporal dynamics are preferentially investigated using electrophysiological techniques such as magnetoencephalography (MEG) and electroencephalography (EEG) that enable direct measurement of neural activity at a high temporal resolution up to the millisecond, as compared to functional magnetic resonance imaging (fMRI) that indirectly measure neural activity via blood oxygenation level dependant (BOLD) brain responses at the second scale. Rapid fluctuations of neural activity captured using electrophysiological measures^9–12^ evidenced rich spatiotemporal dynamics^13–15^ in resting-state networks (RSNs). For instance, Baker and colleagues^16^ showed using hidden Markov modelling (HMM) of band-limited power envelopes of source reconstructed MEG data that activity patterns within RSNs change much more rapidly than previously thought. Indeed, this HMM analysis as well as later studies^17–22^ disclosed discrete transient (100–200 ms) brain states that repeatedly recur over time in RS-related neural activity and correspond to the activation/deactivation of well-known RSNs^14,23,24^. Altogether, HMM studies support the hypothesis that neurocognitive networks adapt to the rapidly changing computational demands of cognitive processing^25^ through rapid reorganization and coordination mechanisms operating at the sub-second time scale^26^. Consequently, the ability of HMM to leverage the excellent temporal resolution of EEG/MEG signals opens interesting prospects to investigate the neural plasticity dynamics underlying ML and its consolidation.

To the best of our knowledge, a single MEG HMM study reported specific changes in movement-related sensorimotor beta-activity during the actual practice of a self-paced sequential visuomotor task^27^. Other MEG studies specifically focusing on the functional connectivity of brain networks over long-time scales (i.e., 5 min) relatedly found sensorimotor-related connectivity predictive of learning levels in a subsequent ML task^28,29^ and offline post-ML changes in mu-beta modulation in the sensorimotor cortex^30^. This indicates that inter- and intraindividual differences in offline minute-long functional network connectivity may reflect learning abilities and brain plasticity mechanisms. Still, these studies do not inform us about the fast (100–300 ms) activation dynamics of RSNs that accompany ML-related plasticity and consolidation. In this context, the present HMM MEG study aimed to test whether intra-individual fast neural dynamics changes from a pre- to a post-learning RS session reflect state-like ML consolidation-related brain plasticity mechanisms. Second, we investigated whether fast transient network dynamics in spontaneous human brain activity during pre-learning RS are predictive of individual performance levels reached in a subsequent learning session and, thus, can be seen as a trait-like marker of individual ML capacity.

## Results

**Figure 1:**
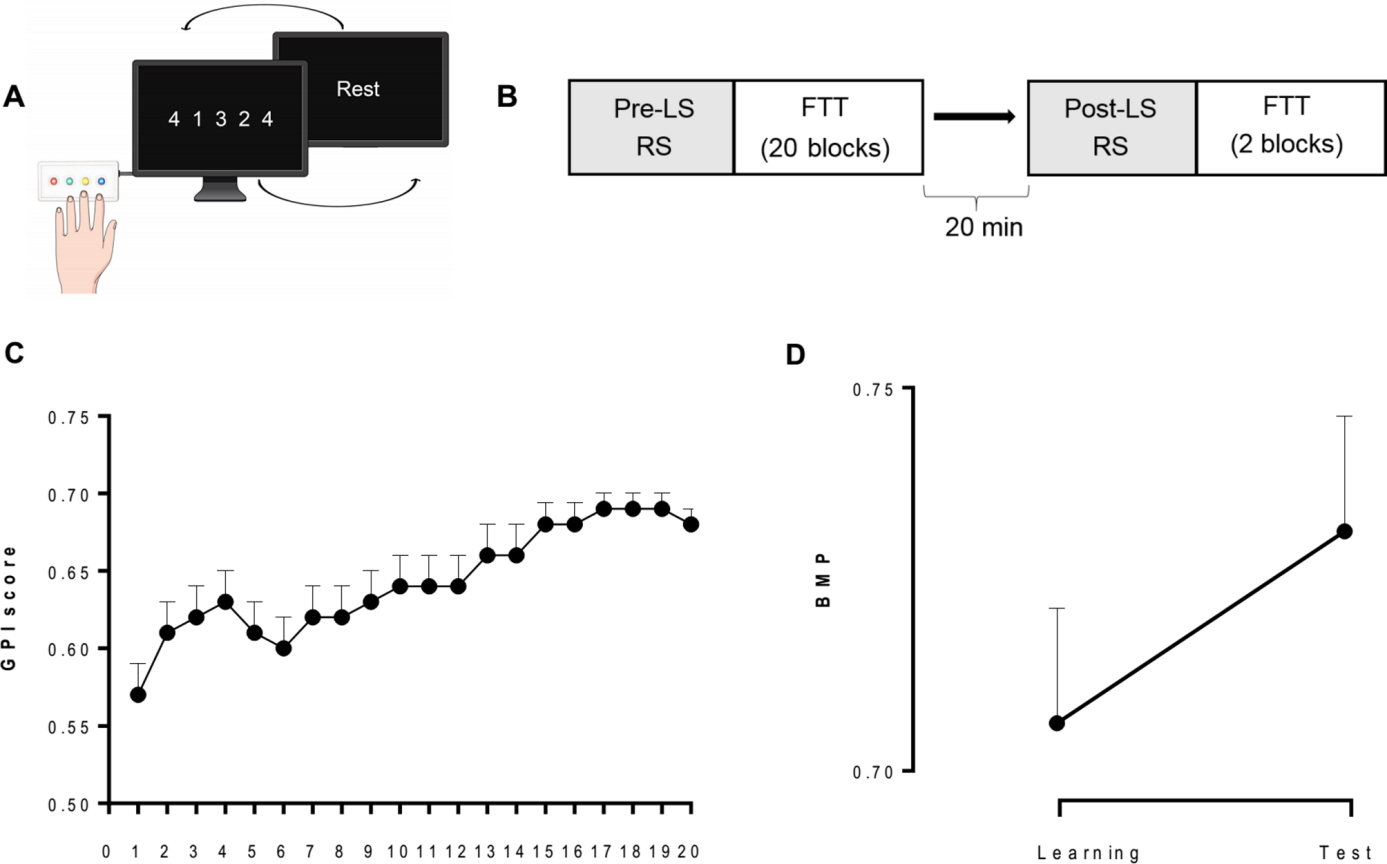
Experimental design and behavioural performance. (**A**) Schematic illustration of the FTT sequence to be reproduced during 30-s experimental blocks designed using Mind The Graph platform, https://mindthegraph.com**. **(**B**) Experimental procedure**:** MEG recorded pre-learning session (LS) resting state (RS; 5 min eyes open), immediately followed by 20 FTT training blocks. After a 20-min break, MEG recorded post-learning RS followed by 2 FTT blocks (Test). (**C**) Global Performance Index (GPI) evolution across the 20 FTT blocks during LS. (**D**) Offline changes from the best motor performance (BMP) at learning to the best performance at testing. Error bars are standard errors. Figures (**C**) and (**D**) were created using GraphPad Prism version 9.1.2, GraphPad Software, San Diego, California USA, www.graphpad.com.

Twenty-seven young healthy participants were trained on a motor sequence learning finger tapping task (FTT^1,4^) (Fig. 1A,B). Brain activity was recorded during pre- and post-learning RS sessions (5-min, eyes open) as well as during learning (data not reported) using a 306 channel MEG Triux neuromagnetometer. Post-learning RS recording was followed by a second FTT test conducted 20 min after the end of ML, i.e., during the boost period.

### Sleepiness and fatigue measures

Prior motor task performance, participants’ drowsiness and fatigue levels were controlled using visual analogue scales of fatigue and sleepiness. There was no significant difference between Learning and Test sessions in sleepiness (t (26) = − 1.16, *p* = 0.26) as well as in fatigue scores (t (26) = -1.58, *p* = 0.13, paired-sampled t-test, Supplementary information, Table 1).

### Motor learning performance

FTT performance was estimated based on the Global Performance Index (GPI) that accounts for speed-accuracy trade-off in the reproduction of motor sequence^31^ (for analyses conducted separately on speed and accuracy measures, see Supplementary Information). During the learning session (LS), performance rapidly improved to reach asymptotic levels at the end of practice (Fig. 1C). For each individual, the best motor performance (BMP) at learning was computed averaging two blocks with the highest GPI scores. The Learning Index (LI; performance improvement from the 2 first learning blocks to BMP level) was significant (LI = 23.1 ± 3.3%; one-sample t-test *t*(26) = 7.0; *p* < 0.001). The offline evolution of performance from BMP at learning to the best score achieved at the retest 20 min later was also significant (Boost effect [BE] = 2.9 ± 0.8%; *t*(26) = 3.7; *p* < 0.001; Fig. 1D).

### Network states of MEG power activation and deactivation

Eight HMM states were inferred from the power envelope of wide-band source-reconstructed MEG RS recordings (MEG signals band-pass filtered in 4–30 Hz, envelopes low-pass filtered at 40 Hz), as in^16^ except that Minimum Norm Estimation (MNE) was used for source projection to better capture the neural activity from posterior midline cortices^20^. The HMM was run on the temporal concatenation of both pre- and post-learning RS sessions across participants. Each HMM state represents a distinct type of bursting with a specific power envelope covariance that recurrently occurs over time^16,32^. Binary time series of state activation/inactivation were estimated using the Viterbi algorithm, which enforces the constraint that two states cannot be active simultaneously^33^. The network topography associated with each state was visualized by mapping the partial correlation of the corresponding activation/inactivation time series with brain envelope signals. These correlation maps allow locating regional power envelope changes (increase/decrease for positive/negative correlation) during state visits. A noteworthy difference between the HMM state and the functional networks, as defined by functional connectivity analysis, is that two states may feature the same networks or split a network into distinct subcomponents due to differences in their fast activation dynamics.

**Figure 2:**
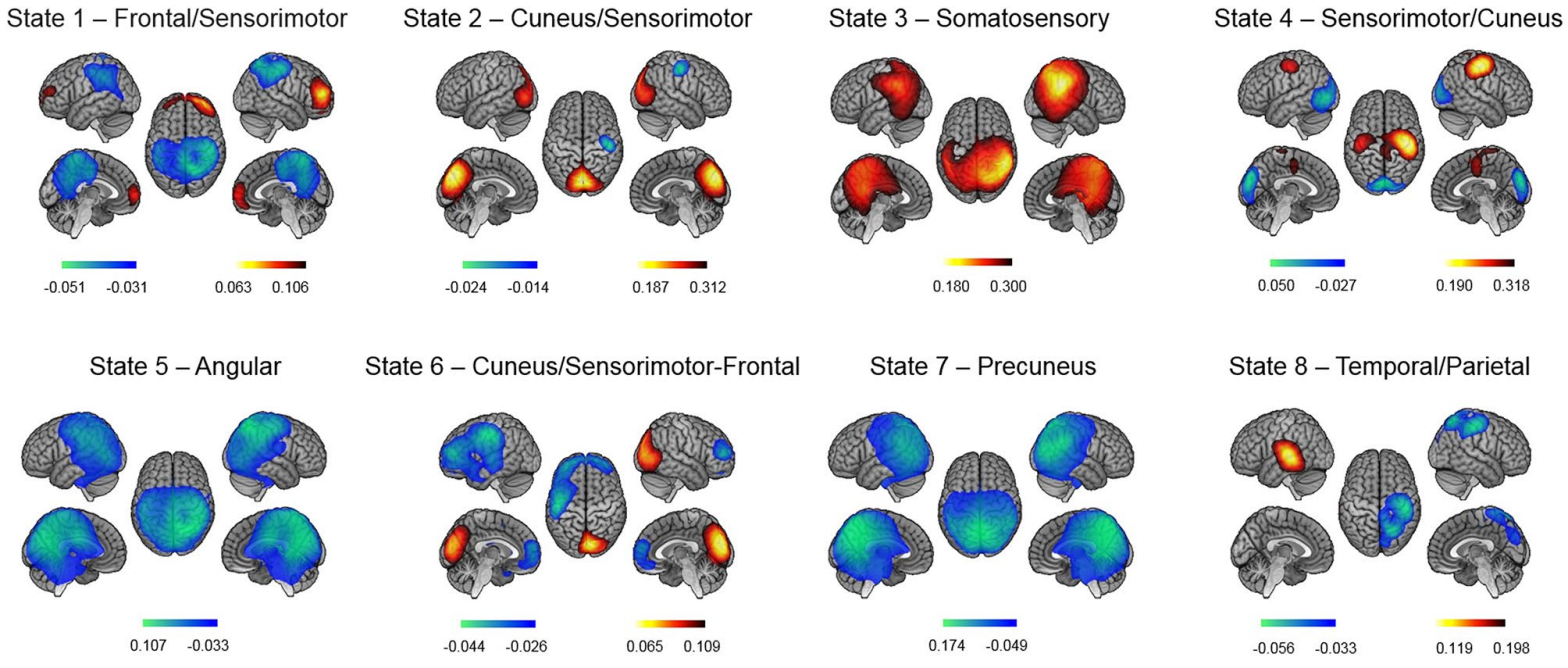
Spatial topographies of HMM transient states computed over pre- and post-learning RS sessions. Red/blue scales indicate positive/negative correlation values between the envelope and the state activation/inactivation time course (i.e., increased/decreased power during one state visit). For visualization purpose, the maps are thresholded between 60 and 100% of the maximum absolute of the partial correlation values. The maps were obtained using MATLAB R2016a, The MathWorks, Inc, https://www.mathworks.com/ and MRIcrone version 1.0.20190902, 2019, Chris Rorden, https://www.nitrc.org.

The HMM state power maps obtained from our dataset disclosed distinctive topographic states related to RSNs (Fig. 2). Frontal/Sensorimotor State 1 featured a network configuration with both increased power over the prefrontal cortex and decreased power bilaterally in sensorimotor cortices. Such anti-correlation between two networks is commonly observed within HMM states^16,20^ and is reminiscent of metastability and dynamical competition in the short-time functional connectivity of RSNs^34^. Cuneus/Sensorimotor State 2 was characterized by increased power peaking at the cuneus and decreased power within the right precentral/postcentral gyri. Somatosensory State 3 was characterized by a bilateral power increase in somatosensory cortices, here without anti-correlation. Sensorimotor/Cuneus State 4 featured the same networks as State 2 but with opposite activation/deactivation patterns, i.e., increased power bilaterally in sensorimotor areas and decreased power in the cuneus. Angular State 5 and Precuneus State 7 were both characterized by decreased power peaking in the right angular gyrus and left precuneus, respectively. Cuneus/Sensorimotor-Frontal State 6 featured increased power in the right cuneus and decreased power in the left sensorimotor cortices and bilateral prefrontal cortex. Finally, Temporal/Parietal State 8 featured increased power in the left auditory cortex and decreased power in the right parietal cortex.

### Effect of motor learning on state temporal characteristics

**Figure 3:**
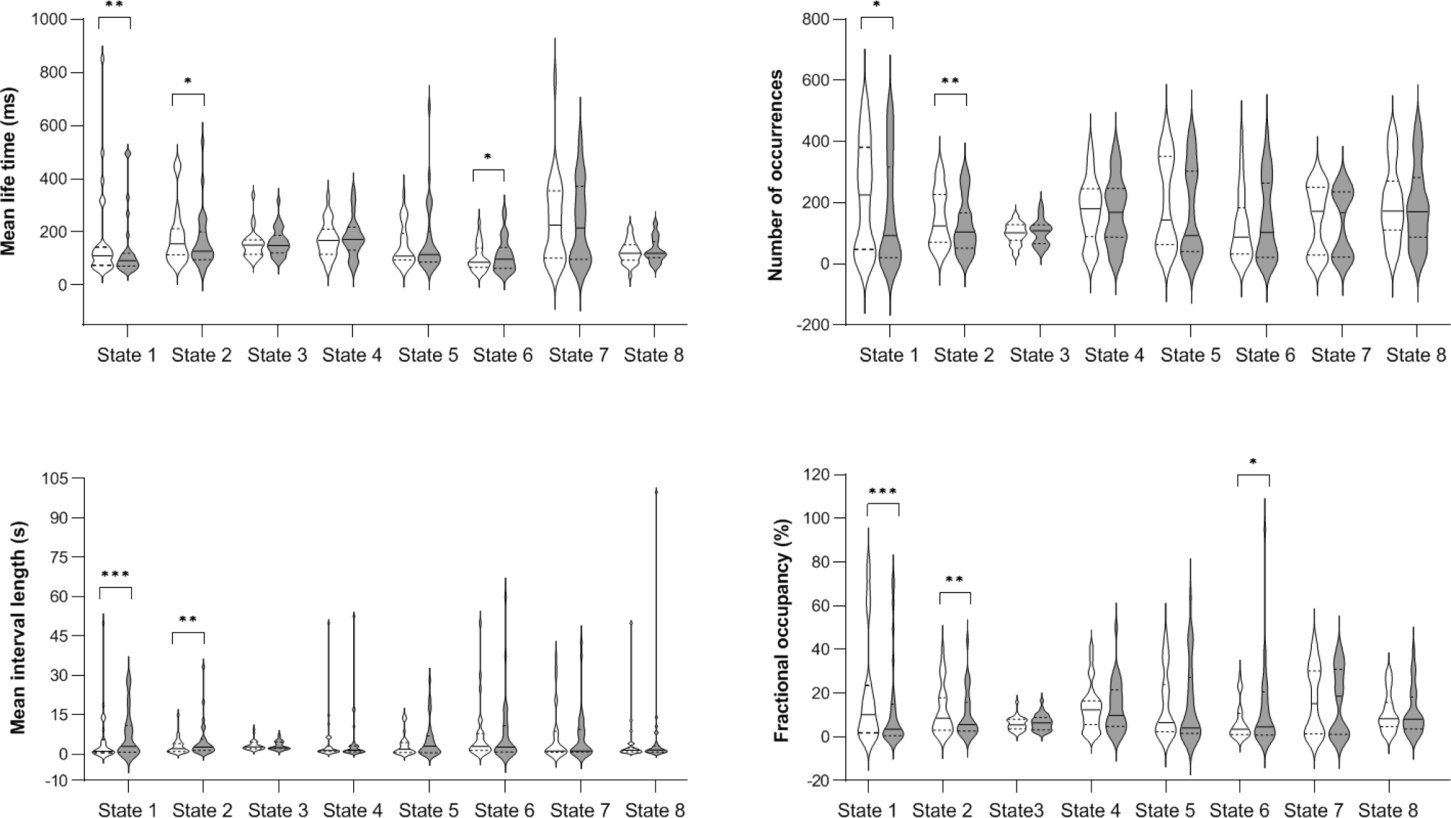
Temporal parameters for HMM states before (Pre, in white) and after (Post, in grey) motor learning [*p < 0.05 (uncorrected); **p < 0.01 and ***p < 0.001 (corrected for 7 independent states)]. Violin plots are shown with the median as a solid line and quartiles as dotted lines. The figure was created in GraphPad Prism version 9.1.2 for Windows, GraphPad Software, San Diego, California USA, www.graphpad.com.

Four state temporal parameters were computed based on the activation/inactivation time course of the 8 states: mean life time (MLT, i.e., the mean time spent in a given state on a single visit), fractional occupancy (FO, i.e., the total fraction of recording time spent in the active state), mean interval length (MIL, i.e., the time elapsed between two visits in the same state) and the number of occurrences (NO, i.e., the entire number of visits in a state)^16,35^. To enable statistical testing, these indices were estimated for each subject and each session (pre- and post-learning) separately by splitting the corresponding state activation/inactivation time series accordingly. Comparisons between pre- and post-learning resting session temporal parameters (Fig. 3) in Frontal-Parietal State 1 revealed significantly decreased MLT (paired sample t-test [Wilcoxon signed-rank test] *W* = 296; *p* < 0.002, multiple comparisons corrected for 7 independent states), FO (*W* = 342; *p* < 0.001) and NO (*W* = 297; *p* < 0.009 uncorrected), as well as significantly increased MIL (*W* = 54; *p* < 0.001) in the post-learning RS session. Likewise, decreased MLT (*W* = 269; *p* < 0.055 uncorrected), FO (*W* = 302; *p* < 0.005) and NO (*W* = 291; *p* < 0.003) and increased MIL (*W* = 75; *p* < 0.005) was found post-learning in Visual-Motor State 2. These two states were therefore visited less frequently, for a shorter duration and at longer intervals after the motor task, suggesting that motor practice may have modulated the associated fast networks. Additionally, a reverse trend was observed (uncorrected) for increased MLT (*W* = 82; *p* < 0.031) and FO (*W* = 91; *p* < 0.017) after motor learning in Cuneus-Postcentral State 6. An additional analysis conducted after removing potential outliers gave essentially similar results (see Supplementary Information, Fig. 3). Comparisons between pre- and post-RS sessions in other states did not reach significance (see Supplementary Information, Table 2).

### Associations between state characteristics and behavioural performance/learning

The findings above suggest that the temporal stability of some fast networks is modulated by motor learning during FTT. However, they do not tell us whether this effect is related to intra-individual learning processes per se, or if they reflect different performance abilities between subjects. To address this question, we searched for associations between the temporal parameters of States 1, 2, and 6 (in pre- and post-learning RS sessions separately) on the one hand, and behavioural indices of motor performance and learning, on the other hand.

**Figure 4:**
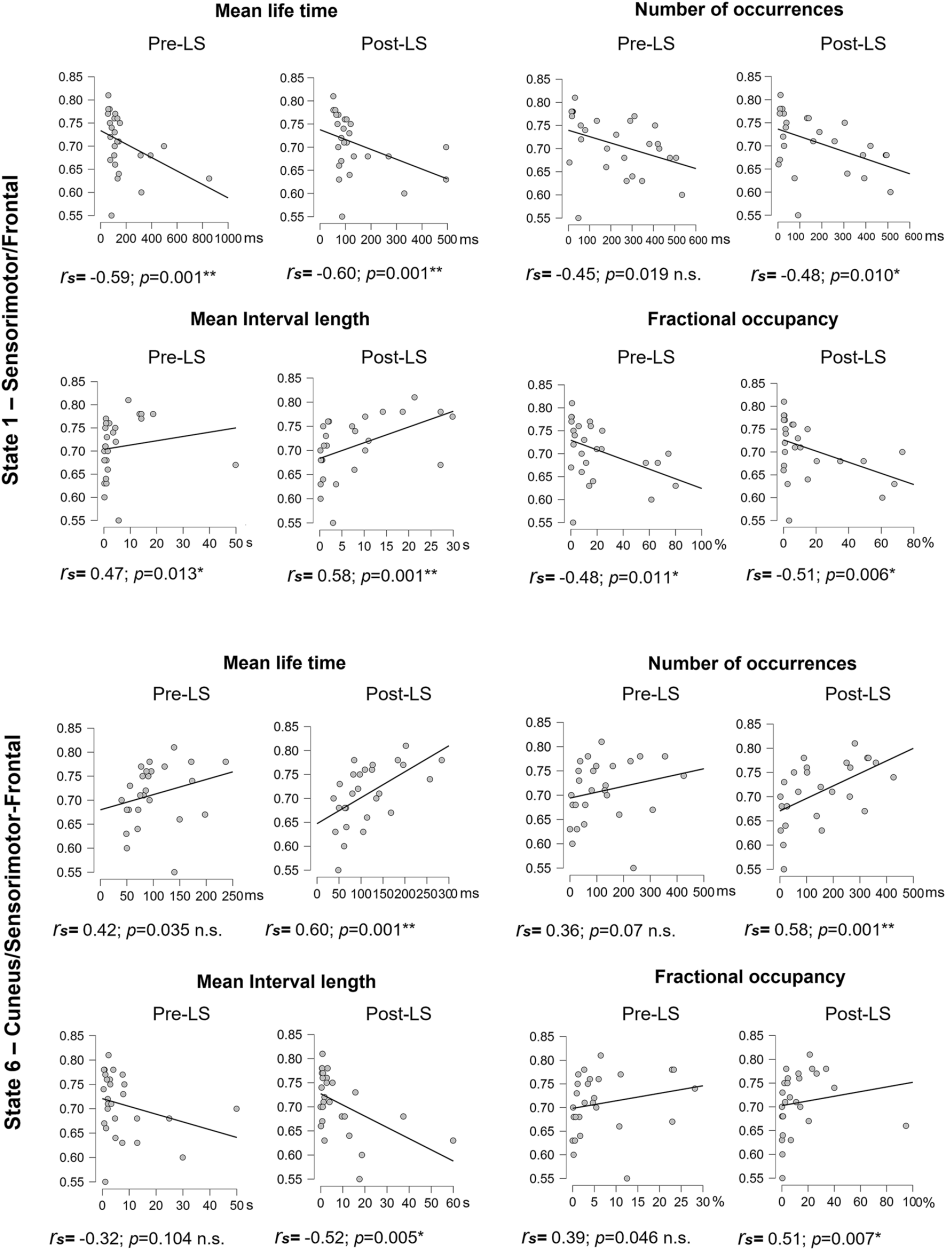
Correlations between best motor performance (BMP) achieved in the Learning session and HMM temporal parameters for States 1 and 6 before (Pre) and after (Post) learning (*p < 0.01 (corrected for 4 HMM temporal parameters); **p < 0.002 (corrected for 4 HMM temporal parameters and 7 states). The figure was created using JASP version 0.14.1, JASP Team (2021), https://jasp-stats.org.

We first considered correlations with the BMP (i.e., the highest motor performance level achieved by an individual during the learning session) (Fig. 4). In State 1, robust associations were identified both in pre- and post-learning sessions. Specifically, in the pre-learning session BMP negatively correlated with MLT (Spearman correlation *r*_*s*_ = − 0.59; *p* < 0.001 corrected for the number of HMM parameters) and FO (*r*_*s*_ = − 0.48; *p* < 0.011) and positively correlated with MIL (*r*_*s*_ = 0.47; *p* < 0.013). A trend for a negative correlation between BMP and NO was also observed (*r*_*s*_ = − 0.45; *p* < 0.019 uncorrected) but did not survive correction for multiple comparisons (corrected significance level *p* < 0.013). In the post-learning session, correlation patterns with BMP were similar (MLT: *r*_*s*_ = − 0.60; *p* < 0.001; MIL: *r*_*s*_ = − 0.51; *p* < 0.006; MIL: *r*_*s*_ = 0.58; *p* < 0.001 and NO: *r*_*s*_ = − 0.48; *p* < 0.010). In State 6, opposite correlation patterns were observed, with a trend in the pre-learning session (MLT *r*_*s*_ = 0.42, *p* < 0.035; FO *r*_*s*_ = 0.39, *p* < 0.046, NO *r*_*s*_ = 0.36; *p* < 0.07) but significant associations in the post-learning session (MLT *r*_*s*_ = 0.60, *p* < 0.001; FO *r*_*s*_ = 0.51, *p* < 0.007; NO *r*_*s*_ = 0.58, *p* < 0.001; MIL *r*_*s*_ = − 0.52, *p* < 0.007). Importantly, in all cases, correlation coefficients did not significantly differ between pre- and post-learning sessions (all *ps* > 0.32), suggesting that the above associations for States 1 and 6 are independent of the offline learning-related reorganization mechanisms. As for State 2, no association was evidenced between BMP and any temporal parameter (see Supplementary Information, Fig. 4; all *r*_*s*_*s* < 0.12, *ps* > 0.54).

**Figure 5:**
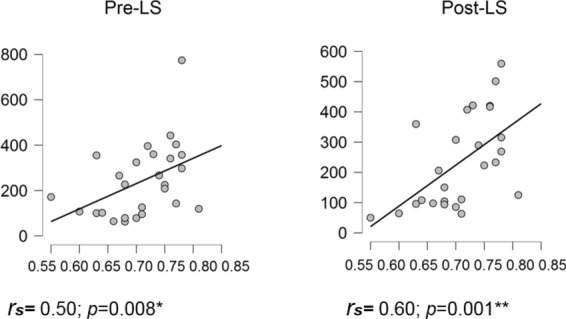
Correlations between best motor performance (BMP) achieved in the Learning session and mean life time (MLT) for State 7 (*p < 0.01, corrected for 4 HMM temporal parameters only; **p < 0.002, corrected for 4 HMM temporal parameters and 7 states). The figure was created using JASP version 0.14.1, JASP Team (2021), https://jasp-stats.org.

Additionally, we performed this same analysis for all HMM states, regardless of the significant changes from pre- to post-learning session. Our results additionally revealed a correlation between BMP and State 7 MLT in the post-learning session *r*_*s*_ = 0.62, *p* < 0.001 (Bonferroni corrected by states by parameters factor = 28) (Fig. 5). Full correlational analysis results can be found in Supplementary Information, Table 2.

A caveat in the analyses above is that best motor performance (BMP) being obtained at the end of learning potentially reflects both motor and sequence learning components and is thus not a mere reflection of individual motor ability. To try dissociating as much as possible motor sequential learning from mere motor execution, we reasoned that the first blocks in the learning session mostly reflect mere individual motor abilities since the sequence to be learned is not yet integrated at the procedural level, whereas with continued practice both motor and sequential components are at play to contribute to performance at the end of learning. Thus, we estimated a baseline (BL) performance as the average of FTT blocks 2 and 3 of the learning session (block 1 was not taken into account in this analysis as due to habituation to the task, variability was high with frequent stops and disruptions in the execution of the sequence), assuming that at that stage participants did not learn yet the sequence, which makes it a potential motor control situation. In a first step, we computed the potential correlation between BL (as a “motor control” condition) and best motor performance (BMP; obtained at the end of learning and reflecting both motor and sequence learning components). Results indicate a strong positive correlation between the two behavioural measures (*r* = 0.81; *p* ≤ 0.001, Pearson’s r) which suggests that performance measured at the end of learning (BMP) is strongly conditioned by the participant’s initial motor ability (BL). In a second step, we computed correlations between motor BL condition and state parameters measures, which essentially resulted in similar patterns than correlations with BMP as can be seen in Supplementary Table 6. This indicates that correlation between performance and state parameters are at least to a large extent likely conditioned by the individual’s motor ability more than sequential learning.

In the next step, we investigated potential associations between HMM temporal parameters and behavioural indices specific to the evolution of motor learning rather than mere motor ability. Correlations with LI (which reflects the intra-individual evolution of performance within the learning session) did not highlight any robust associations (all *ps* > 0.02 uncorrected) and correlation coefficients were not significantly different between pre- and post-learning sessions. Likewise, associations between LI and pre- to post-practice changes in state temporal characteristics were nonsignificant (all *ps* > 0.03 uncorrected, Supplementary Information, Table 4). Finally, we searched for associations between offline performance changes (i.e., BE; from the best performance during learning to the test session 20 min later, see Supplementary Information Table 5). State 3 mean life time (MLT) in the post-learning session positively correlated with the boost effect (BE), rs = 0.48, p < 0.012 (Supplementary Information, Fig. 6). However, after applying a more stringent correction for multiple comparisons by factor 28 [number of temporal parameters and number of states (4 × 7)], this correlation did not survive the statistical threshold. Finally, we examined whether motor learning is reflected in changes to the pattern of transitions between the states, i.e., we computed a transition probability matrix^16^ and compared the transitions before and after learning (Supplementary Information, Fig. 8). However, no result passed the statistical threshold (corrected for 8 states × 7 probabilities).

## Discussion

This study investigated using HMM of MEG power envelope signals whether fast transient networks in spontaneous human brain activity are predictive of individual motor capabilities, and whether post-learning changes in fast neural dynamics reflect learning-related brain plasticity mechanisms. Results show an association between motor performance levels achieved at learning and the temporal stability of Frontal/Sensorimotor State 1 and of Cuneus/Sensorimotor-Frontal State 6, both during pre- and post-motor learning RS sessions, suggesting a trait-like relationship between the spontaneous organisation of fast recurrent brain networks involved in motor learning and motor ability. Lack of correlations with behavioural indices of learning-related brain plasticity suggests that such transient dynamics may not reflect state-like neural changes underlying motor learning and consolidation, at least within the short time scale of early consolidation mechanisms.

We hypothesised that post-learning changes in neural dynamics would reflect offline brain plasticity memory consolidation mechanisms initiated during a short time window after the end of learning^2,4^. Although behavioural data evidenced, as expected, performance improvement during learning (LI) and an offline boost (BE) 20 min after the end of learning, none of these behavioural indices of learning-related brain plasticity was associated with any state dynamics. This lack of association might be due to the fact that plastic changes supporting motor learning simultaneously recruit local and distant brain areas in a hierarchy of temporal scales ranging from sub-second to hours. Functional MRI^8^ and MEG^28,30^ studies highlighted motor learning- and memory consolidation-related neural networks using resting-state functional connectivity (rsFC) measures. The fundamental assumption behind these methods is that the temporal correlation of spontaneous brain signals among spatially distributed brain regions creates temporally stable (at least for several seconds) functional networks. The HMM probes the fast activation dynamics of these networks, and particularly their temporal stability, at much shorter time scales, which may prevent capturing robust plastic changes occurring at slower time scales. It has been suggested that the proportion of time spent by a subject in each brain network and metastates (hierarchically organized brain states) is a consistent subject-specific measure^36^, genetically determined and exhibiting significant relationships with cognitive traits. Thus, neuroplastic changes may occur within certain adaptation boundaries in each specific brain. Additionally, neuroplasticity might boost the development of neural connections that facilitate performance speed and accuracy, without a considerable influence on the temporal stability of the network as a whole^37^, thus making consolidation changes concealed from the HMM states of MEG power activity.

These results raise the question of whether observed HMM configurations are ingrained, stable patterns of neural activity for a given individual, i.e., a neuronal trait, or may be modulated by experience and environmental demands, i.e., neuronal states reflecting brain plasticity mechanisms potentially linked to offline learning and consolidation processes. Noticeably, previous HMM studies mostly used between-group designs to evidence differences between populations^20,38,39^. To the best of our knowledge, the present study is the first to investigate the evolution of HMM parameters in a within-subject design during pre- and post-motor learning resting-state sessions, considering motor performance levels and learning-related changes. In our study, HMM inferred 8 transient recurring states from MEG resting-state power envelope data, featuring quite similar spatial and temporal parameters than in previous reports^17,20,40^. Quantitative temporal parameters measuring HMM state stability and recurrence were modified from pre- to post-learning sessions in States 1, 2, and 6, which might be due to the task in between the two RS sessions, alternatively to the time elapsed between the two RS acquisitions. Considering the former explanation, the destabilization of Frontal/Sensorimotor State 1 after motor learning could be explained by the automatization of the practiced motor act, eventually requiring at the same time lower levels of cognitive control (i.e., less activation of the frontal control-executive network) and higher involvement of motor-related areas (i.e., less deactivation of the sensorimotor network) contributing to motor memory consolidation. Such argument would find support in prior research showing that learning a motor skill relies in the early stage on the activity of executive prefrontal cortices, which in turn informs lower motor levels (i.e., premotor and primary motor cortices^41^). With the progress of learning, cortical activity in the frontal lobe diminishes following an anterior to posterior axis^42–46^, which enables a transition from executive to motor levels of control and consequently, a reduction in central resource demands. However, our correlational analyses showed that the best motor performance (BMP) achieved during the learning session was strongly correlated with State 1 temporal parameters *both* in the pre- and post-RS sessions, without differences between the two sessions. This rather suggests the existence of a stable, trait-like neural activity pattern associated with individual motor performance ability, a conclusion additionally supported by the fact that we found similar correlation patterns when running correlations with performance in the two first blocks (baseline, BL) of the learning session during which the sequence to be learned is not yet integrated at the procedural level. In other words, one may surmise that increased power in frontal executive networks together with decreased power in sensorimotor areas might be an important pre-setting for successful execution of a motor task and/or indicate good individual motor abilities. On the other hand, this could be a result of previous experience and prolonged training over the individual’s development in motor tasks such as sport, playing video games, fine motor skills hobbies, etc.

On another note, no correlation was found between State 2 and BMP, although HMM parameters differed between sessions, suggesting that pre- to post-learning changes in State 2 dynamics were independent of motor-related effects. At variance, there was a trend for a positive association between BMP and State 6 parameters in pre-learning RS session. In both States 2 and 6, power increased in cuneus areas and decreased in sensorimotor cortices upon state activation. Functional relationships between cuneus and somatosensory areas have been previously suggested^47^, with the proposal that correlation between these brain areas in the resting-state contributes to the anti-correlated activity reported between the default-mode network (metabolically activated at rest^48^) and other brain networks activated in the context of tasks that require visual/sensory processing. Although one study found increased functional connectivity within sensorimotor and visual RSNs shortly after sequence learning^8^, no correlations with performance were reported. State 6 also disclosed deactivation pattern in the frontal executive network opposite to that in State 1, which might be key to explain the directionality of associations between motor performance levels and their temporal parameters. Indeed, better performance levels were negatively correlated with temporal parameters in State 1, and positively in State 6, in line with a potential disengagement of the executive network after motor learning. The trend for stabilization of State 6 post-learning, and thus the fact that its motor-related areas spend more time being deactivated, is also consistent with decreased activity in M1 at late skill learning^49^ and automatized^45^ stages. Although modified power within sensorimotor cortices in States may be viewed as indicative of learning-related plastic changes, our correlation results do not support this interpretation, as associations between brain activity patterns and motor performance are similar before and after the learning episode. Hence, our current results are in favour of the hypothesis that fast brain dynamics reflect a possibly intrinsic (i.e., task independent), trait-like brain architecture associated with the ability to perform a motor task.

A potential limitation in the interpretation of our results is the difficulty to warrant a clear-cut dissociation between motor learning and motor execution with a task like the FTT in which participants repeatedly reproduce as fast as possible for 30 s per block pre-defined, explicitly known sequences of finger movement. Indeed, if participants are asked as a control condition to perform a “simpler” basic sequential tapping task (e.g. 1–2–3–4–1–2–3–4–…), there is still a sequential component to be optimized. If on the other hand, they are asked to perform single finger tapping (or all fingers simultaneously), the co-articulation motor movement components are completely lost and the two tasks then differ by much more than the motor (sequential) learning component. Theoretically, it might be possible to estimate the impact of sequence learning besides the motor component by asking participants to learn a second, different sequence and compute proactive interference effects of the first sequence on the acquisition of the second one, like was done for motor adaptation tasks^50^, but this was not implemented in the current protocol. To address at least in part this issue, we calculated a baseline (BL) performance index as the average of the first FTT blocks of the learning session, assuming that at that stage participants did not learn the sequence yet, which makes it a potential motor control situation. As reported above, our results not only disclosed high correlation coefficients between BL and BMP behavioural value, but also that correlation analyses between HMM parameters and BL scores essentially result in similar patterns than with BMP (see Supplementary Information, Table 6), supporting our assumption that correlations between performance and state parameters are at least to a large extent conditioned by the individual’s motor ability more than sequential learning, even if we acknowledge that we cannot completely rule out a contribution of the latter.

On another note, it has been shown that changing the number of states in HMM analyses can alter the burst characteristics of each state^51^. Therefore, the choice of the number of states (which has to be predefined in HMM) may impact the observed results. We opted here for an a priori number of eight states, both based on existing literature and our own lab’s experience. The determination of an optimal number of states is important in order to both avoid surplus information and not to miss the activity of interest. Indeed, a too low number of states (e.g., 3) might lead to miss a potential dissociation between motor learning and memory consolidation-related activity. On the other hand, a too high number of states may split the motor-related activity into several, more or less redundant states with low temporal characteristics. For instance, the mean life time parameter would be shorter in those states since distributed over a few states, as shown by Seedat et al.^51^. In the seminal Baker et al.^16^ HMM-envelope paper, the authors suggested that the optimal number of states can be estimated via the model with the lowest free energy value, and ended up choosing 8 states as a good trade-off between richness and redundancy. Additionally, in a recent paper from our group^52^, the number of states was reduced to 6 and the state topographies were quite similar to those obtained with 8 states^20,39^, suggesting that the amount of information obtained for 6 and 8 states is relatively similar. Although we cannot exclude potential limitations due to our a priori choice of using 8 states, we opted to use a standard a priori number rather conducting parallel analyses manipulating the number of states, which would have led us to the problem of selecting the best analysis parameters based on their output in terms of results, which is circular.

To sum up, our results suggest a robust trait-like relationship between interindividual variations in motor performance capability and fast transient neural dynamics during the resting state. Missing associations with brain plasticity-related learning and memory consolidation behavioural parameters suggest that fast transient RS dynamics may not be modulated by learning experience and reflect state-like neural plasticity mechanisms, at least on the short consolidation timescale of the present experiment.

## Methods

### Participants and procedure

Thirty-four participants were recruited for this experiment, according to estimated sample size (a medium size effect in a within-subjects design study using a two-tailed t-test, with effect size 0.5, significance level 0.05, and power of 0.8). All subjects were young and healthy right-handed individuals, non-musician or professional typist who gave written informed consent to participate in this study which was approved by the CUB Hôpital Erasme Ethics Committee (Ref: P2016/553; CCB: B406201630539). However, 7 participants had to be removed after the preliminary analysis: 3 datasets were discarded due to poor motor performance; 1 dataset had a corrupted MEG signal and 3 participants were identified as outliers in Hidden Markov model analysis due to aberrant, extreme values. All in all, the data of 27 participants (11 females; mean age = 23.4 ± 2.7 years, range 18–29) are reported. All methods were performed in accordance with relevant guidelines and regulations. Female participants were tested during the second week of their menstrual cycle to avoid hormonal influence on motor learning abilities^53^. Caffeine-containing drinks and food, soda, and any other stimulants were prohibited 12 h before testing.

Upon arrival at the laboratory, participants were prepared for the MEG recording. They then underwent in the MEG scanner a first 5-min resting-state (RS) session (pre-learning) in a seated posture with the eyes open focused on a fixation cross on the wall. Then, participants filled the visual analogue scales (VAS) of fatigue and sleepiness in order to identify potential effects of drowsiness and fatigue. Immediately after, they were trained on a 5-elements Finger Tapping Task (FTT^4^; adapted from^54^). In this task (Fig. 1A), each finger corresponded to one digit (from 1 = little finger to 4 = index) and participants were instructed to continuously reproduce a 5-element sequence of finger movements (4–1–3–2–4) as fast and accurately as possible with their non-dominant hand for 30 s (1 block). The sequence to reproduce was permanently displayed on the computer screen during execution. Participants were familiarized with the task during 2 FTT demo blocks then underwent the experimental learning session (LS) with twenty 30-s FTT blocks with 20-s breaks in between blocks while being in the MEG scanner. Right after the task, participants were allowed to leave the scanner chair and move within the room. At the end of the 20-min break, they filled in VAS and performed a second MEG RS session (post-learning). Finally, performance gains from the end of learning were evaluated by performing two FTT blocks (Test session; Fig. 1B).

### Behavioural indices for motor performance and learning

Motor performance during the FTT was estimated for each block by computing a Global Performance Index (GPI) that takes into account both speed and accuracy^31^. Based on the GPI scores, we identified best motor performance (BMP) reached by each subject as the average of the two LS blocks with the highest GPI scores, which reflects a contribution of both motor and sequential. We also estimated a baseline (BL) performance as the average of FTT blocks 2 and 3 of the learning session (block 1 was not taken into account in this analysis as due to initial habituation to the task, variability was high with frequent stops and disruptions in the execution of the sequence), assuming that at that stage participants did not learn yet the sequence, which makes it a potential motor control situation. To measure the evolution of performance during the learning session, we computed the Learning Index (LI) as the percentage change in GPI scores from BL to BMP. Finally, we estimated offline changes in performance from the end of learning to the test session (i.e., boost effect) as the percentage change from BMP to the best GPI score achieved after the post-learning testing.

### Neuroimaging data acquisition

MEG data acquisition was performed using a 306 channel whole-scalp MEG system (Triux, MEGIN, Helsinki, Finland) located inside a light-weight magnetically shielded room (Maxshield, MEGIN, Helsinki, Finland) at the CUB Hôpital Erasme (Brussels, Belgium). Participants’ head position was tracked continuously within the MEG helmet by four head tracking coils. Coils’ position and about 300 head points were determined following the anatomical fiducials with an electromagnetic tracker (Fastrak, Polhemus, Colchester, Vermont, USA). We applied an online analog band-pass filter in the range of 0.1–330 Hz for all recordings and digitized the signal at 1 kHz sampling rate. Participant’s high-resolution 3D T1-weighted cerebral magnetic resonance images (MRIs) were acquired after the MEG recordings on a 1.5 T MRI scanner (Intera, Philips, The Netherlands).

### Data pre-processing

The temporal signal space separation method^55^ was applied offline to the continuous MEG data to minimize external magnetic interference and head movements corrections (Maxfilter v2.1, MEGIN, Finland). Then, data were filtered (offline band-pass filter: 0.1–45 Hz) and an independent component analysis (FastICA algorithm with dimension reduction to 30 components, hyperbolic tangent nonlinearity function)^56^ was applied for visual inspection. Independent components corresponding to cardiac, ocular and system artifacts were rejected by regressing their time course out of the full-rank data. To proceed with source reconstruction, the MEG forward models were estimated based on the participants’ 3D T1-weighted cerebral MRI, anatomically segmented using FreeSurfer software (version 6.0; Martinos Center for Biomedical Imaging, Massachusetts, USA). The MEG and MRI coordinate systems were co-registered via three anatomical fiducials points (nasion and auricular) for primary head position estimation and the head-surface points for manual refinement (MRIlab, MEGIN Data Analysis Package 3.4.4, MEGIN, Helsinki, Finland). A volumetric and regular 5-mm source grid was constructed in the Montreal Neurological Institute (MNI) template MRI and non-linearly deformed onto each participants’ MRI with the Statistical Parametric Mapping Software (SPM12, Wellcome Centre for Neuroimaging, London, UK). Finally, the three-dimensional MEG forward model associated with this source space was estimated using a one-layer Boundary Element Method as executed in the MNE-C suite.

Source projection of MEG data was then based on Minimum Norm Estimation (MNE)^57^. The noise covariance matrix was estimated form 5-min empty room MEG recordings spatially filtered using signal space separation method^55^ and temporally filtered between 0.1 and 45 Hz. The MNE regularization parameter was fixed using the consistency condition derived in^58^. Three-dimensional dipole time courses were projected on their direction of maximum variance and their Hilbert envelope signal was extracted using the Hilbert transform.

### Hidden Markov model dynamic analysis

The analysis followed the pipeline described by^16,35^ implemented in GLEAN (https://github.com/OHBA-analysis/GLEAN). The main difference in our implementation is that MNE is used as an inverse model rather than the Beamformer, as MNE allows the investigation of RSNs states related to the DMN, and in particular, states involving posterior midline cortices (i.e., precuneus and posterior cingulate cortex)^20,39^. The number of transient states was set to 8 for consistency with previous MEG power envelope HMM studies^16,20,35,39^. The 8-state HMM was inferred from the wide-band filtered (4–30 Hz) source envelope signals. Envelope data were downsampled at 10 Hz using a moving-window average with 75% overlap (100 ms wide windows, sliding every 25 ms), resulting in effective downsampling at 40 Hz, demeaned, normalized by the global variance, and temporally concatenated across participants to design a group-level HMM analysis and across the two RS sessions in order to identify network states common to both the pre- and post-learning sessions (for further discussion on this strategy, see, e.g.^20^). The concatenated envelopes were then pre-whitened and reduced to 40 principal components. Finally, the HMM algorithm^33,59^ was repeatedly run on this dataset 10 times (to account for different initial parameters and retain the model with the lowest free energy) to determine states classifying different power envelope covariance patterns. The Viterbi algorithm was used to decode the binary signals of temporally exclusive state activation/inactivation. Based on these signals, four state temporal parameters were estimated: MLT (mean duration of time intervals of active state), FO (entire fraction of time of the active state), MIL (mean duration of time intervals of inactive state) and NO (total number of state visits). These indices were estimated separately for each subject and session by "de-concatenating" the state activation time series. State power maps were obtained as a result of the partial correlation between HMM state activation/deactivation time series and the concatenated source envelope signals, which assesses state-specific power changes upon state activation.

### Statistical contrasts and correlation analyses with HMM state temporal parameters

The comparison between pre- and post-learning HMM states’ temporal parameters was assessed using paired-sampled Wilcoxon signed-rank. P-values were Bonferroni corrected for multiple comparisons using a factor 7, i.e., the number of independent HMM states (knowledge of the activation of 7 out of 8 HMM states fully determines the activation of the last HMM state due to the temporal exclusion constraint implemented in the Viterbi algorithm)^20^. For HMM states disclosing a significant pre-to-post learning effect, Spearman's rank correlation analyses investigated the relationship between their temporal parameters in pre- and post-learning sessions and behavioural indices. Non-parametric tests were favoured due to higher robustness against outliers, which sometimes arise among HMM state temporal parameters when, e.g., one or a few subjects scarcely visit one state. Results were considered significant at p < 0.05 corrected for multiple comparisons by factor 4, i.e., the number of HMM temporal parameters considered. Finally, we used Bayesian statistics to provide an estimate of the likelihood of the null hypothesis (null hypothesis significance testing; NHST) in the case of non-significant results (results reported as Supplementary Information material).

## Supplementary Information

Supplementary Information.
