# Supplementary material for: Resting state fast brain dynamics predict interindividual variability in motor performance
Source: Sci Rep. 2022 Mar 29;12:5340. doi: 10.1038/s41598-022-08767-z (PMC8964712; doi:10.1038/s41598-022-08767-z)
Supplement: Supplementary file 1 — Supplementary Information. [file 41598_2022_8767_MOESM1_ESM.docx]

**Resting state fast brain dynamics predict interindividual variability in motor performance**

*Supplementary information*

Liliia Roshchupkina^1,2,3^, Vincent Wens^3,4^ , Nicolas Coquelet ^3,4^, Xavier de Tiege^1,3,4^ , Philippe Peigneux^1,2^

^1^ UR2NF—Neuropsychology and Functional Neuroimaging Research Unit affiliated at CRCN - Centre for Research in Cognition and Neurosciences, avenue F.D. Roosevelt 50, Bruxelles 1050, Belgium.

^2^ UNI—ULB Neuroscience Institute, Université Libre de Bruxelles (ULB), avenue F.D. Roosevelt 50, Bruxelles 1050, Belgium.

^3^ Laboratoire de Cartographie fonctionnelle du Cerveau (LCFC), Université libre de Bruxelles (ULB), Brussels, Belgium.

^4^ Department of Functional Neuroimaging, Service of Nuclear Medicine, CUB Hôpital Erasme, Université libre de Bruxelles (ULB), Brussels, Belgium

**I. Visual Analogue Scales of Fatigue**

Prior motor task performance, participants’ drowsiness and fatigue levels were controlled using the Visual Analogue Scales of Fatigue (VASF). There was no significant difference between Learning and Test sessions in sleepiness scores (t(26) = -1.16, *p* = .26 ) as well in fatigue (t(26) = -1.58, *p* = .13, paired-sampled t-test). The descriptive statistics results are displayed in Table S1 below.

**Table S1. Visual analog scales (VAS) scores as percentage of drowsiness and fatigue**

| **Session** | **Measure** | **Mean (%)** | **SD (%)** | **Min (%)** | **Max (%)** |
| --- | --- | --- | --- | --- | --- |
| **LS** | Sleepiness | 22.9 | 15.6 | 3.0 | 62.6 |
|  | Fatigue | 19.7 | 16.7 | 0.0 | 68.0 |
| **Test** | Sleepiness | 25.1 | 16.1 | 3.0 | 62.0 |
|  | Fatigue | 24.7 | 15.8 | 4.5 | 72.0 |

**II. FTT speed**

To facilitate comparisons with prior FTT studies, we computed the evolution of performance using speed and accuracy measures separately. Speed was computed as the number of correct triplets (three-element chunks belonging to the trained sequence) generated per 30-sec block. We used triplets but not the whole 5-element sequence since humans are sensitive to the temporal context set by previous stimuli up to three elements during motor sequence learning ^1,^ ^2^. Later, in the course of learning, a sequence becomes hierarchically organized into chunks and thus represents a single memory unit. Then, units are progressively assembled in a complete sequence ^2,3^. Figure 1.A displays speed FTT performance during the Learning session. The Learning index (LI) was estimated as performance improvement from the 2 first learning blocks to the best speed level (mean of two best LS blocks): LI = 59.2 +/-6.6%; one-sample t-test t(26) = 9.0; *p* < .001. The offline performance improvement from the best speed achieved at learning to the best score reached at the retest (Test session) was also significant: Boost effect [BE] = 10.2+/-2.0%; one-sample t-test t(26) = 5.0; *p* < .001 (Figure 1.B).


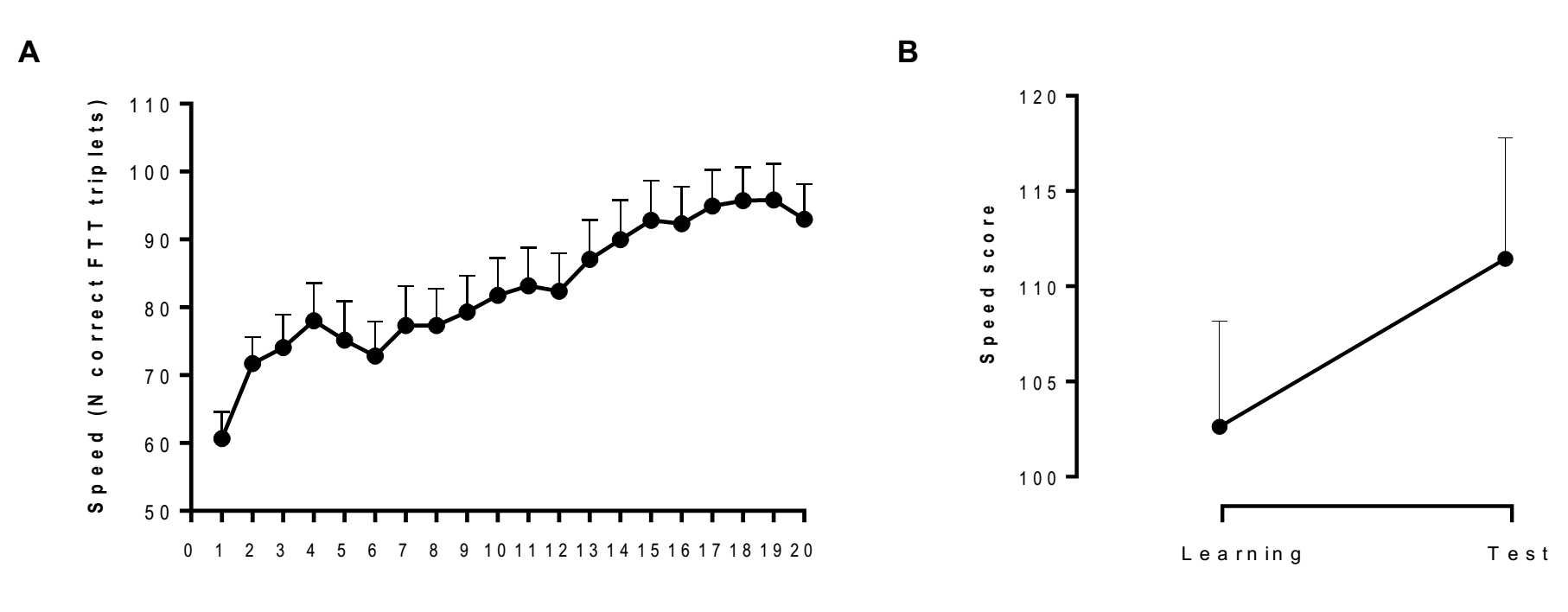


**Figure 1.** Speed FTT performance. **A.** Speed measures during the Learning session. Each dot represents the number of correctly generated FTT triplets within a 30-sec trained block. **B.** Offline speed improvement from best speed achieved in the Learning session to best speed reached during the Test session. Error bars represent standard errors. The figures were created using GraphPad Prism version 9.1.2, GraphPad Software, San Diego, California USA, [www.graphpad.com](http://www.graphpad.com).

**III. FTT accuracy**

Accuracy was computed as the percentage of correctly executed triplets out of the total number of triplets performed per 30-sec block during the Learning session. Figure 2.A represents the accuracy measures across twenty 30-sec blocks. The Learning index (LI) calculated as the change from the 2 first learning blocks to the best accuracy level showed during LS was not significant: LI = -1.3 +/- 0.9%; one-sample t-test (26) = -1.4; *p* = 0,17. The offline change in accuracy from the maximum value achieved at learning to the best score reached at the retest was significant with a slight decrease at the retest: Boost effect [BE] = -2.0 +/-0.5%; one-sample t-test t(26) = -4.3; *p* < .001 (Figure 2.B).


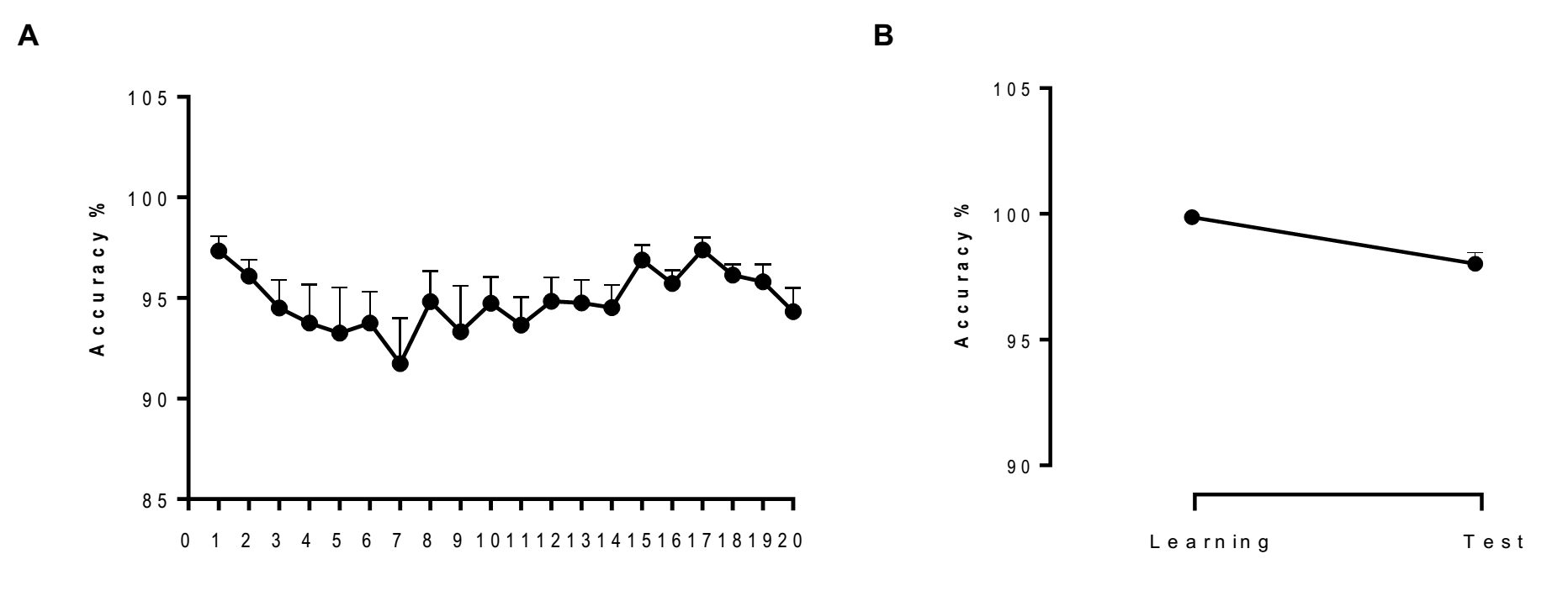


**Figure 2.** FTT accuracy. **A.** Accuracy measures during the Learning session. Black dots stand for % of correct triplets generated within the 30-sec FTT block. **B.** Offline changes from the best speed performance at learning to the best performance at testing. Black bars represent standard errors. The figures were created using GraphPad Prism version 9.1.2, GraphPad Software, San Diego, California USA, [www.graphpad.com](http://www.graphpad.com).

**IV. Effect of motor learning on state temporal characteristics**

To reveal possible effect of motor learning we computed four state temporal parameters based on the activation/inactivation time course of the 8 states: mean life time (MLT, i.e., the mean time spent in a given state on a single visit), fractional occupancy (FO, i.e., the total fraction of recording time spent in the active state), mean interval length (MIL, i.e., the time elapsed between two visits in the same state) and the number of occurrences (NO, i.e., the entire number of visits in a state). These indices were estimated for each subject and each session (pre- and post-learning) separately by splitting the corresponding state activation/inactivation time series accordingly and compared by paired sample t-test [Wilcoxon signed-rank test]. The results are displayed in a table below (Table 2).

**Table 2. Comparison of HMM temporal parameters between pre- and post-learning sessions**

| **Measure** | **State** | **Wilcoxon signed-rank** | **P value** |
| --- | --- | --- | --- |
| Mean life time  (MLT) | S1 | 296.000 | 0.002* |
|  | S2 | 269.000 | 0.06 |
|  | S3 | 167.000 | 0.61 |
|  | S4 | 130.000 | 0.16 |
|  | S5 | 137.000 | 0.22 |
|  | S6 | 82.000 | 0.03* |
|  | S7 | 175.000 | 1.00 |
|  | S8 | 149.000 | 0.35 |
| Fractional occupancy (FO) | S1 | 342.000 | < .001** |
|  | S2 | 302.000 | 0.005** |
|  | S3 | 148.000 | 0.336 |
|  | S4 | 133.000 | 0.185 |
|  | S5 | 168.000 | 0.628 |
|  | S6 | 91.000 | 0.017 |
|  | S7 | 215.000 | 0.546 |
|  | S8 | 185.000 | 0.934 |
| Mean interval length  (MIL) | S1 | 50.000 | 0.001** |
|  | S2 | 75.000 | 0.006** |
|  | S3 | 199.500 | 0.810 |
|  | S4 | 206.000 | 0.446 |
|  | S5 | 144.000 | 0.290 |
|  | S6 | 213.000 | 0.347 |
|  | S7 | 145.000 | 0.446 |
|  | S8 | 136.500 | 0.328 |
| Number of occurrences  (NO) | S1 | 297.500 | 0.009* |
|  | S2 | 291.000 | 0.003** |
|  | S3 | 164.000 | 0.556 |
|  | S4 | 165.000 | 0.572 |
|  | S5 | 241.000 | 0.216 |
|  | S6 | 121.500 | 0.107 |
|  | S7 | 224.500 | 0.218 |
|  | S8 | 212.500 | 0.580 |

**Note.** Comparison of HMM temporal parameters between pre- and post-learning sessions in Wilcoxon signed-rank t-test (*p*-values (**p - not corrected; **p- corrected by factor 7).*

To control for the robustness of reported effects, we repeated the comparison analyses between pre- and post-learning resting session temporal parameters (shown in main text Figure 3) after excluding potential outliers defined as 3^rd^ quartile + 3*interquartile interval (IQI) for the upper range and 1^st^ quartile – 3*IQI for the lower range. Outlier values were replaced with the group’s mean. This novel analysis showed similar results, with slight differences in p-values as indicated on the graph below (Figure 3).


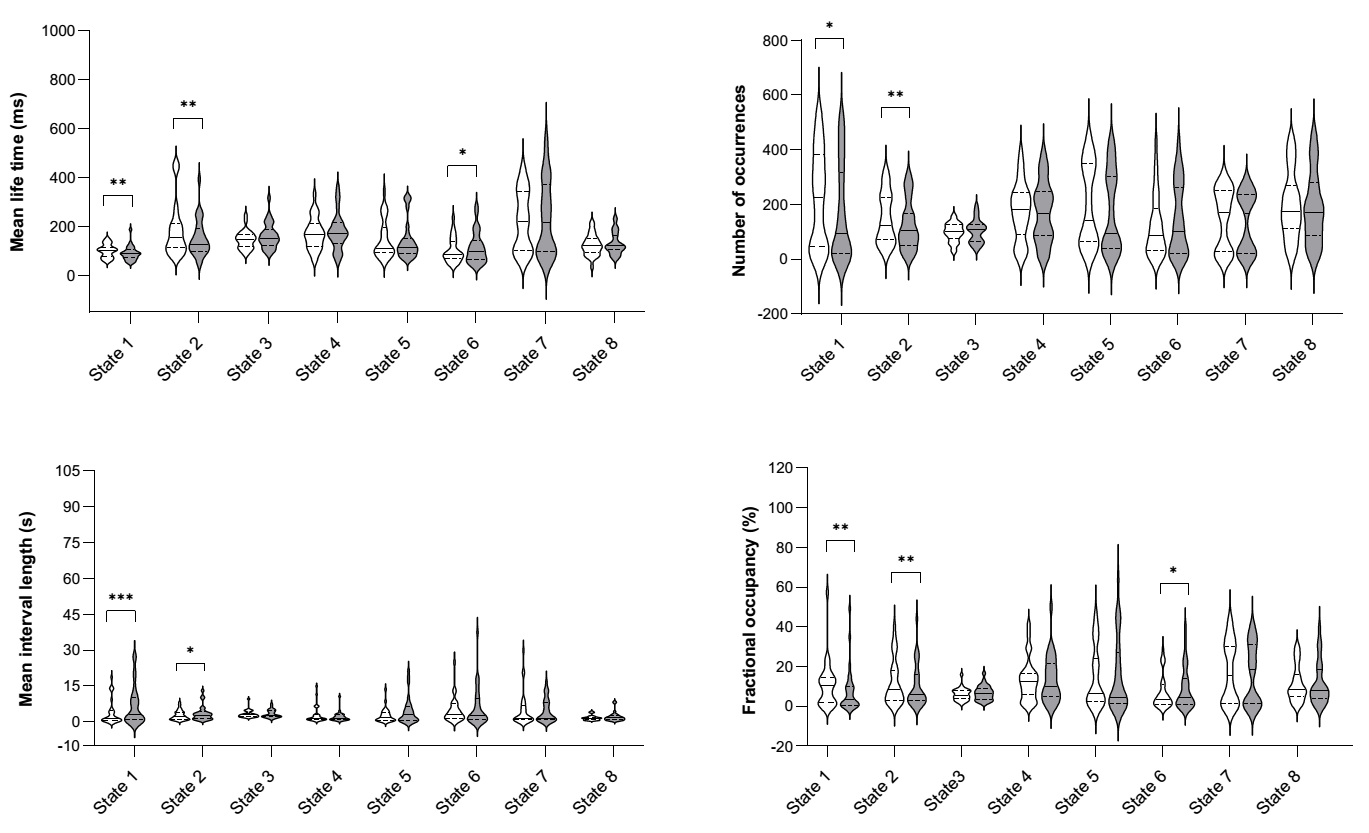


**Figure 3.** Temporal parameters for HMM states before (Pre, in white) and after (Post, in grey) motor learning (^*^ *p* < .05 (uncorrected); ** *p* < .01 and *** *p* < .001 (corrected for 7 independent states)), after removal of potential outliers (3rd quartile + 3*interquartile interval (IQI) for the upper range and 1st quartile – 3*IQI for the lower range). Violin plots are shown with the median as a solid line and quartiles as dotted lines. The figure was created in GraphPad Prism version 9.1.2 for Windows, GraphPad Software, San Diego, California USA, [www.graphpad.com](http://www.graphpad.com).

**V. Correlational analyses between HMM parameters and BMP**

For assessing the relationship between motor performance and HMM temporal parameters, we performed a correlational analysis between BMP and HMM temporal parameters. Figure 4 represents the correlational results for State 2 – Cuneus/Sensorimotor (Spearman correlation *r_s_s* < .12, *ps* > .54) that was found modified in post- as compared to-re-learning session (see main text).


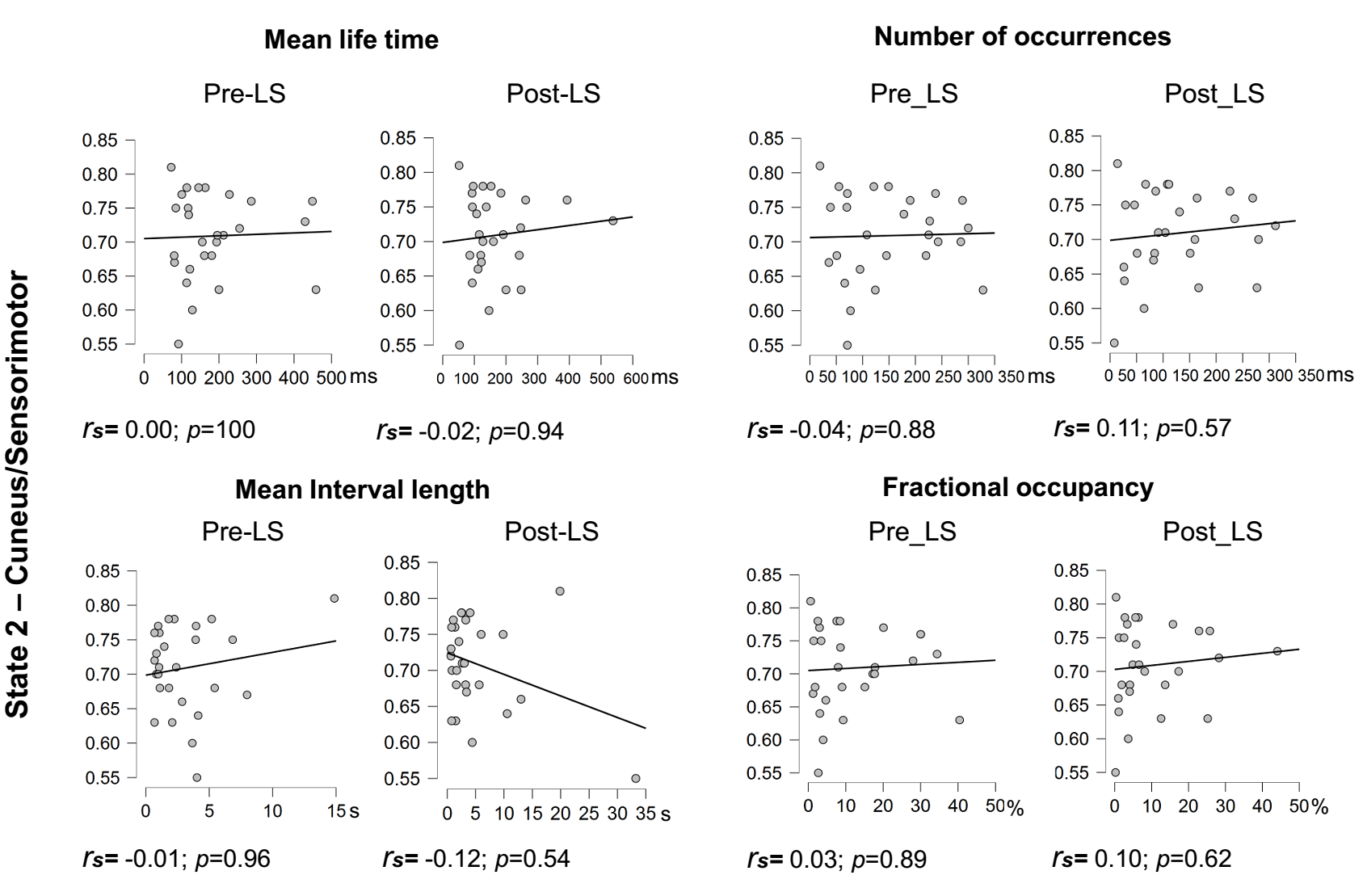


**Figure 4.** Correlations between best motor performance (BMP) achieved in the Learning session and HMM temporal parameters for State 2 – Cuneus/Sensorimotor before (Pre) and after (Post) learning. The figure was created using JASP version 0.14.1, JASP Team (2021), <https://jasp-stats.org>.

Additionally, and for the sake of completeness, we report here all correlations statistics between behavioural indices (BMP, Learning Index (LI) and Boost Effect (BE)) for all 8 HMM states. We also provide Bayesian analyses estimating of the likelihood of the null hypothesis (null hypothesis significance testing; NHST, BF01). As can be seen in Tables 3, 4 and 5 below, results in States others than the ones analysed in the main paper (i.e., S1, S2 and S6 in which HMM parameters differ between pre- and post-learning session) were not consistent. More precisely, only few correlations survived after applying a liberal correction by factor 4 (i.e., only the number of HMM temporal parameters). In this context, mean life time (MLT) in State 7 positively correlated with BMP in both pre- and post-learning sessions, r_s_ = 0.50, p < .008 and r_s_ = 0.62, p < .001 (reported in the main text). Additionally, State 7 fractional occupancy (FO) in the post-learning session correlated with BMP: r_s_ = 0.48, p < .012 (corrected by number of HMM temporal parameters; Figure 5). State 3 mean life time (MLT) in the post-learning session positively correlated with the boost effect (BE), r_s_ = 0.48, p < .012 (Figure 6). Finally, the correlation between BE and State 3 proportional change (post/pre) in FO was found r_s_ = 0.54, p = .004 (Figure 7). Still, when applying a more stringent correction for multiple comparisons by factor 28 (i.e., number of temporal parameters and number of states (4x7)), only one correlation survived the threshold, namely, BMP and State 7 mean life time (MLT) in the post-learning session r_s_ = 0.62, p < .001.

**Table 3.** **Correlations between Best Motor Performance (BMP) and HMM temporal parameters**

| **Measure** | **State** | **Session** | **Correlation coefficient**  **Spearman’s rho** | **Spearman’s**  **p values** | **Correlation coefficient**  **Kendall’s**  **tau-b** | **Bayes Factor (BF01)** |
| --- | --- | --- | --- | --- | --- | --- |
| Mean life time  (MLT) | S1 | Pre | -0.59 | <0.01 | -0.44 | 0.03 |
|  |  | Post | -0.60 | <0.01 | -0.45 | 0.02 |
|  |  | Post/Pre | 0.09 | 0.67 | 0.06 | 3.64 |
|  | S2 | Pre | 0.00 | 1.00 | 0.02 | 3.40 |
|  |  | Post | -0.02 | 0.94 | -0.01 | 4.04 |
|  |  | Post/Pre | -0.07 | 0.73 | -0.10 | 3.19 |
|  | S3 | Pre | 0.36 | 0.07 | 0.30 | 0.41 |
|  |  | Post | 0.37 | 0.12 | 0.22 | 1.13 |
|  |  | Post/Pre | -0.01 | 0.95 | 0.03 | 3.95 |
|  | S4 | Pre | -0.05 | 0.80 | -0.04 | 3.90 |
|  |  | Post | -0.11 | 0.56 | -0.10 | 3.10 |
|  |  | Post/Pre | -0.15 | 0.45 | -0.11 | 2.95 |
|  | S5 | Pre | -0.03 | 0.88 | -0.03 | 3.94 |
|  |  | Post | -0.17 | 0.39 | -0.10 | 3.15 |
|  |  | Post/Pre | -0.28 | 0.17 | -0.20 | 1.44 |
|  | S6 | Pre | 0.42 | 0.04 | 0.35 | 0.20 |
|  |  | Post | 0.60 | <0.01 | 0.46 | 0.02 |
|  |  | Post/Pre | 0.34 | 0.08 | 0.27 | 0.61 |
|  | S7 | Pre | 0.50 | 0.01 | 0.45 | 0.26 |
|  |  | Post | 0.60 | <0.01 | 0.46 | 0.03 |
|  |  | Post/Pre | 0.17 | 0.39 | 0.13 | 2.60 |
|  | S8 | Pre | 0.43 | 0.03 | 0.30 | 0.47 |
|  |  | Post | 0.42 | 0.03 | 0.30 | 0.43 |
|  |  | Post/Pre | -0.10 | 0.63 | -0.10 | 3.19 |
| Fractional  occupancy  (FO) | S1 | Pre | -0.48 | 0.01 | -0.36 | 0.14 |
|  |  | Post | -0.51 | 0.01 | -0.40 | 0.07 |
|  |  | Post/Pre | -0.28 | 0.17 | -0.18 | 1.70 |
|  | S2 | Pre | -0.03 | 0.89 | 0.01 | 4.03 |
|  |  | Post | 0.10 | 0.62 | 0.08 | 3.45 |
|  |  | Post/Pre | 0.12 | 0.55 | 0.08 | 3.45 |
|  | S3 | Pre | 0.34 | 0.09 | 0.25 | 0.82 |
|  |  | Post | 0.36 | 0.06 | 0.25 | 0.85 |
|  |  | Post/Pre | 0.01 | 0.97 | 0.03 | 3.95 |
|  | S4 | Pre | 0.01 | 0.95 | 0.01 | 4.03 |
|  |  | Post | -0.14 | 0.49 | -0.13 | 2.71 |
|  |  | Post/Pre | -0.18 | 0.38 | -0.12 | 2.75 |
|  | S5 | Pre | -0.18 | 0.36 | -0.11 | 2.91 |
|  |  | Post | -0.23 | 0.25 | -0.15 | 2.31 |
|  |  | Post/Pre | -0.31 | 0.12 | -0.21 | 2.75 |
|  | S6 | Pre | 0.39 | 0.05 | 0.30 | 0.41 |
|  |  | Post | 0.51 | 0.01 | 0.37 | 0.13 |
|  |  | Post/Pre | 0.32 | 0.10 | 0.24 | 0.90 |
|  | S7 | Pre | 0.45 | 0.02 | 0.33 | 0.26 |
|  |  | Post | 0.48 | 0.01 | 0.35 | 0.19 |
|  |  | Post/Pre | 0.08 | 0.72 | 0.05 | 3.79 |
|  | S8 | Pre | 0.42 | 0.03 | 0.29 | 0.53 |
|  |  | Post | 0.37 | 0.06 | 0.28 | 0.53 |
|  |  | Post/Pre | 0.04 | 0.83 | 0.00 | 4.04 |
| Mean interval  length  (MIL) | S1 | Pre | 0.47 | 0.01 | 0.36 | 0.16 |
|  |  | Post | 0.58 | <0.01 | 0.45 | 0.44 |
|  |  | Post/Pre | 0.26 | 0.19 | 0.17 | 1.95 |
|  | S2 | Pre | -0.01 | 0.97 | -0.01 | 4.03 |
|  |  | Post | -0.12 | 0.54 | -0.08 | 3.37 |
|  |  | Post/Pre | -0.20 | 0.32 | -0.18 | 1.76 |
|  | S3 | Pre | -0.40 | 0.04 | -0.27 | 0.62 |
|  |  | Post | -0.38 | 0.05 | -0.24 | 0.79 |
|  |  | Post/Pre | -0.17 | 0.41 | -0.16 | 2.17 |
|  | S4 | Pre | 0.04 | 0.83 | 0.03 | 3.93 |
|  |  | Post | 0.14 | 0.50 | 0.11 | 2.96 |
|  |  | Post/Pre | 0.16 | 0.42 | 0.11 | 2.96 |
|  | S5 | Pre | 0.22 | 0.27 | 0.15 | 2.25 |
|  |  | Post | 0.26 | 0.20 | 0.17 | 1.90 |
|  |  | Post/Pre | 0.29 | 0.15 | 0.25 | 0.85 |
|  | S6 | Pre | -0.32 | 0.10 | -0.24 | 0.85 |
|  |  | Post | -0.52 | 0.01 | -0.39 | 0.09 |
|  |  | Post/Pre | -0.12 | 0.55 | -0.07 | 3.54 |
|  | S7 | Pre | -0.48 | 0.01 | -0.35 | 0.19 |
|  |  | Post | -0.39 | 0.05 | -0.26 | 0.73 |
|  |  | Post/Pre | -0.06 | 0.77 | -0.08 | 3.50 |
|  | S8 | Pre | -0.41 | 0.03 | -0.28 | 0.53 |
|  |  | Post | -0.37 | 0.06 | -0.28 | 0.53 |
|  |  | Post/Pre | -0.10 | 0.64 | -0.17 | 2.00 |
| Number of  Occurrences  (NO) | S1 | Pre | -0.45 | 0.02 | -0.35 | 0.19 |
|  |  | Post | -0.49 | 0.01 | -0.39 | 0.08 |
|  |  | Post/Pre | -0.33 | 0.09 | -0.23 | 1.1 |
|  | S2 | Pre | -0.03 | 0.88 | -0.02 | 4.01 |
|  |  | Post | 0.11 | 0.57 | 0.08 | 3.37 |
|  |  | Post/Pre | 0.25 | 0.21 | 0.20 | 1.45 |
|  | S3 | Pre | 0.40 | 0.04 | 0.27 | 0.61 |
|  |  | Post | 0.38 | 0.05 | 0.24 | 0.90 |
|  |  | Post/Pre | 0.15 | 0.45 | 0.13 | 2.60 |
|  | S4 | Pre | -0.07 | 0.75 | -0.04 | 3.85 |
|  |  | Post | -0.15 | 0.45 | -0.14 | 2.50 |
|  |  | Post/Pre | -0.12 | 0.57 | -0.06 | 3.63 |
|  | S5 | Pre | -0.23 | 0.25 | -0.16 | 2.10 |
|  |  | Post | -0.26 | 0.19 | -0.17 | 1.91 |
|  |  | Post/Pre | -0.23 | 0.26 | -0.16 | 2.14 |
|  | S6 | Pre | 0.36 | 0.07 | 0.27 | 0.63 |
|  |  | Post | 0.58 | <0.01 | 0.41 | 0.06 |
|  |  | Post/Pre | 0.42 | 0.03 | 0.33 | 0.27 |
|  | S7 | Pre | 0.33 | 0.09 | 0.26 | 0.76 |
|  |  | Post | 0.36 | 0.06 | 0.26 | 0.75 |
|  |  | Post/Pre | 0.09 | 0.66 | 0.07 | 3.60 |
|  | S8 | Pre | 0.40 | 0.04 | 0.27 | 0.65 |
|  |  | Post | 0.36 | 0.06 | 0.27 | 0.63 |
|  |  | Post/Pre | 0.16 | 0.43 | 0.09 | 3.23 |

**Note.** Correlations between Best Motor Performance (BMP) and HMM temporal parameters at Pre- and Post-learning sessions, and between BMP and proportional changes in HMM parameter values from pre- to post-learning session (Post/Pre), for all States. Spearman’s Rho and p-value. Bayesian correlations (Kendall’s tau-b) and Bayes Factors BF01 are provided as indications of potential support for the null hypothesis (Supportive = BF > 3; Inconclusive = 0.33 < BF < 3; Against = BF < 0.33).


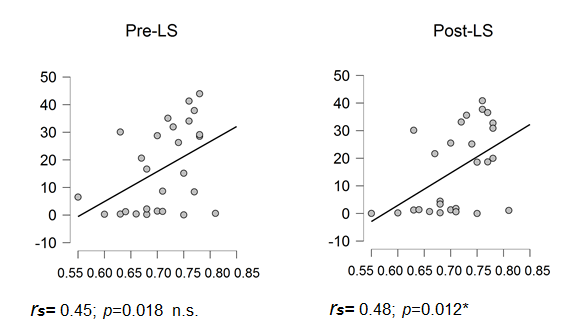


**Figure 5.** Correlations between the best motor performance (BMP) achieved in the Learning session and fractional occupancy (FO) for State 7 *(*p < .013, corrected for 4 HMM temporal parameters only).* The figure was created using JASP version 0.14.1, JASP Team (2021), <https://jasp-stats.org>.

**Table 4. Correlations between Learning Index (LI) and HMM temporal parameters**

| **Measure** | **State** | **Session** | **Correlation coefficient**  **Spearman’s rho** | **Spearman’s**  **p values** | **Correlation coefficient**  **Kendall’s**  **tau-b** | **Bayes Factor (BF01)** |
| --- | --- | --- | --- | --- | --- | --- |
| Mean life time  (MLT) | S1 | Pre | 0.08 | 0.70 | 0.04 | 3.90 |
|  |  | Post | 0.20 | 0.31 | 0.13 | 2.59 |
|  |  | Post/Pre | 0.22 | 0.28 | 0.16 | 2.08 |
|  | S2 | Pre | -0.33 | 0.08 | -0.23 | 1.02 |
|  |  | Post | -0.36 | 0.07 | -0.26 | 0.71 |
|  |  | Post/Pre | 0.01 | 0.94 | 0.03 | 3.94 |
|  | S3 | Pre | -0.19 | 0.33 | 0.04 | 3.90 |
|  |  | Post | -0.15 | 0.46 | -0.12 | 2.74 |
|  |  | Post/Pre | 0.08 | 0.70 | 0.03 | 3.95 |
|  | S4 | Pre | 0.03 | 0.89 | 0.05 | 3.80 |
|  |  | Post | -0.01 | 0.99 | 0.01 | 4.02 |
|  |  | Post/Pre | 0.08 | 0.70 | -0.11 | 2.95 |
|  | S5 | Pre | 0.27 | 0.18 | 0.18 | 1.76 |
|  |  | Post | 0.28 | 0.16 | 0.18 | 1.71 |
|  |  | Post/Pre | 0.05 | 0.82 | -0.20 | 1.44 |
|  | S6 | Pre | 0.07 | 0.72 | 0.05 | 3.74 |
|  |  | Post | -0.20 | 0.32 | -0.15 | 2.34 |
|  |  | Post/Pre | -0.29 | 0.15 | -0.22 | 1.14 |
|  | S7 | Pre | -0.34 | 0.09 | -0.25 | 0.89 |
|  |  | Post | -0.37 | 0.06 | -0.29 | 0.53 |
|  |  | Post/Pre | 0.03 | 0.87 | 0.13 | 2.60 |
|  | S8 | Pre | 0.01 | 0.99 | 0.02 | 4.01 |
|  |  | Post | -0.06 | 0.78 | -0.02 | 4.01 |
|  |  | Post/Pre | -0.08 | 0.67 | -0.10 | 3.19 |
| Fractional  occupancy  (FO) | S1 | Pre | 0.04 | 0.85 | 0.03 | 3.97 |
|  |  | Post | 0.03 | 0.89 | 0.02 | 4.00 |
|  |  | Post/Pre | 0.13 | 0.52 | 0.08 | 3.42 |
|  | S2 | Pre | -0.27 | 0.18 | -0.15 | 2.34 |
|  |  | Post | -0.44 | 0.02 | -0.34 | 0.21 |
|  |  | Post/Pre | -0.30 | 0.13 | -0.22 | 1.23 |
|  | S3 | Pre | 0.06 | 0.78 | 0.05 | 3.83 |
|  |  | Post | -0.11 | 0.59 | -0.06 | 3.68 |
|  |  | Post/Pre | -0.16 | 0.42 | -0.11 | 3.01 |
|  | S4 | Pre | 0.13 | 0.52 | 0.12 | 2.74 |
|  |  | Post | 0.04 | 0.85 | 0.04 | 3.90 |
|  |  | Post/Pre | -0.07 | 0.71 | -0.06 | 3.71 |
|  | S5 | Pre | 0.33 | 0.90 | 0.24 | 0.95 |
|  |  | Post | 0.19 | 0.34 | 0.13 | 2.54 |
|  |  | Post/Pre | -0.05 | 0.82 | -0.05 | 3.83 |
|  | S6 | Pre | 0.17 | 0.39 | 0.13 | 2.54 |
|  |  | Post | -0.15 | 0.46 | -0.11 | 3.02 |
|  |  | Post/Pre | -0.18 | 0.36 | -0.12 | 2.70 |
|  | S7 | Pre | -0.31 | 0.12 | -0.23 | 1.09 |
|  |  | Post | -0.37 | 0.06 | -0.27 | 0.66 |
|  |  | Post/Pre | -0.18 | 0.37 | -0.11 | 2.92 |
|  | S8 | Pre | 0.07 | 0.73 | 0.03 | 3.97 |
|  |  | Post | 0.07 | 0.74 | 0.05 | 3.80 |
|  |  | Post/Pre | -0.06 | 0.77 | 0.01 | 4.04 |
| Mean interval length  (MIL) | S1 | Pre | -0.07 | 0.71 | -0.05 | 3.75 |
|  |  | Post | -0.12 | 0.55 | -0.08 | 3.39 |
|  |  | Post/Pre | -0.08 | 0.69 | -0.04 | 3.88 |
|  | S2 | Pre | 0.21 | 0.29 | 0.11 | 2.93 |
|  |  | Post | 0.43 | 0.03 | 0.32 | 0.28 |
|  |  | Post/Pre | 0.42 | 0.03 | 0.34 | 0.21 |
|  | S3 | Pre | -0.09 | 0.67 | -0.05 | 3.80 |
|  |  | Post | 0.14 | 0.48 | 0.11 | 2.98 |
|  |  | Post/Pre | 0.31 | 0.12 | 0.22 | 1.17 |
|  | S4 | Pre | -0.23 | 0.25 | -0.20 | 1.58 |
|  |  | Post | -0.05 | 0.82 | -0.03 | 3.96 |
|  |  | Post/Pre | 0.13 | 0.51 | 0.09 | 3.26 |
|  | S5 | Pre | -0.32 | 0.10 | -0.23 | 1.05 |
|  |  | Post | -0.21 | 0.31 | -0.14 | 2.49 |
|  |  | Post/Pre | 0.04 | 0.86 | 0.03 | 3.94 |
|  | S6 | Pre | -0.18 | 0.38 | -0.13 | 2.54 |
|  |  | Post | 0.13 | 0.53 | 0.09 | 3.22 |
|  |  | Post/Pre | 0.25 | 0.21 | 0.18 | 1.80 |
|  | S7 | Pre | 0.26 | 0.19 | 0.19 | 1.57 |
|  |  | Post | 0.33 | 0.10 | 0.23 | 1.02 |
|  |  | Post/Pre | 0.23 | 0.25 | 0.15 | 2.22 |
|  | S8 | Pre | -0.05 | 0.79 | -0.01 | 4.02 |
|  |  | Post | -0.05 | 0.79 | -0.04 | 3.85 |
|  |  | Post/Pre | 0.02 | 0.93 | -0.03 | 3.94 |
| Number of  Occurrences  (NO) | S1 | Pre | 0.04 | 0.86 | 0.02 | 4.00 |
|  |  | Post | 0.05 | 0.80 | 0.04 | 3.88 |
|  |  | Post/Pre | 0.13 | 0.50 | 0.09 | 3.34 |
|  | S2 | Pre | -0.20 | 0.32 | -0.10 | 3.17 |
|  |  | Post | -0.41 | 0.03 | -0.29 | 0.48 |
|  |  | Post/Pre | -0.40 | 0.04 | -0.30 | 0.40 |
|  | S3 | Pre | 0.04 | 0.84 | 0.03 | 3.97 |
|  |  | Post | -0.10 | 0.63 | -0.08 | 3.46 |
|  |  | Post/Pre | -0.28 | 0.16 | -0.19 | 1.56 |
|  | S4 | Pre | 0.22 | 0.27 | 0.19 | 1.67 |
|  |  | Post | 0.06 | 0.75 | 0.04 | 3.85 |
|  |  | Post/Pre | -0.15 | 0.47 | -0.10 | 3.16 |
|  | S5 | Pre | 0.32 | 0.11 | 0.23 | 1.09 |
|  |  | Post | 0.18 | 0.37 | 0.11 | 2.89 |
|  |  | Post/Pre | -0.10 | 0.61 | -0.09 | 3.34 |
|  | S6 | Pre | 0.19 | 0.36 | 0.14 | 2.44 |
|  |  | Post | -0.23 | 0.26 | -0.16 | 2.05 |
|  |  | Post/Pre | -0.30 | 0.13 | -0.22 | 1.19 |
|  | S7 | Pre | -0.29 | 0.15 | -0.20 | 1.44 |
|  |  | Post | -0.38 | 0.06 | -0.28 | 0.53 |
|  |  | Post/Pre | -0.18 | 0.36 | -0.14 | 2.44 |
|  | S8 | Pre | 0.06 | 0.75 | 0.02 | 4.01 |
|  |  | Post | 0.05 | 0.82 | 0.04 | 3.85 |
|  |  | Post/Pre | -0.04 | 0.83 | 0.01 | 4.04 |

**Note.** Correlations between Learning Index (LI) and HMM temporal parameters at Pre- and Post-learning sessions, and between LI and proportional changes in HMM parameter values from pre- to post-learning session (Post/Pre), for all States. Spearman’s Rho and p-value. Bayesian correlations (Kendall’s tau-b) and Bayes Factors BF01 are provided as indications of potential support for the null hypothesis (Supportive = BF > 3; Inconclusive = 0.33 < BF < 3; Against = BF < 0.33).

**Table 5. Correlations between Boost Effect (BE) and HMM temporal parameters**

| **Measure** | **State** | **Session** | **Correlation coefficient**  **Spearman’s rho** | **Spearman’s**  **p values** | **Correlation coefficient**  **Kendall’s tau-b** | **Bayes Factor (BF01)** |
| --- | --- | --- | --- | --- | --- | --- |
| Mean life time  (MLT) | S1 | Pre | 0.12 | 0.56 | 0.07 | 3.58 |
|  |  | Post | 0.06 | 0.77 | 0.05 | 3.80 |
|  |  | Post/Pre | -0.06 | 0.76 | -0.01 | 4.02 |
|  | S2 | Pre | 0.06 | 0.76 | 0.06 | 3.72 |
|  |  | Post | -0.03 | 0.86 | -0.02 | 3.99 |
|  |  | Post/Pre | -0.28 | 0.16 | -0.18 | 1.80 |
|  | S3 | Pre | 0.13 | 0.53 | 0.10 | 3.08 |
|  |  | Post | 0.48 | 0.01 | 0.34 | 0.22 |
|  |  | Post/Pre | 0.45 | 0.02 | 0.30 | 0.40 |
|  | S4 | Pre | -0.42 | 0.03 | -0.30 | 0.42 |
|  |  | Post | -0.19 | 0.32 | -0.11 | 2.89 |
|  |  | Post/Pre | 0.33 | 0.10 | 0.21 | 1.27 |
|  | S5 | Pre | -0.09 | 0.66 | -0.07 | 3.51 |
|  |  | Post | -0.02 | 0.94 | -0.03 | 3.97 |
|  |  | Post/Pre | 0.10 | 0.61 | 0.07 | 3.58 |
|  | S6 | Pre | 0.09 | 0.67 | 0.05 | 3.76 |
|  |  | Post | 0.09 | 0.65 | 0.06 | 3.72 |
|  |  | Post/Pre | 0.16 | 0.44 | 0.10 | 3.07 |
|  | S7 | Pre | -0.01 | 0.97 | 0.03 | 3.96 |
|  |  | Post | -0.11 | 0.56 | -0.06 | 3.61 |
|  |  | Post/Pre | -0.08 | 0.69 | -0.08 | 3.50 |
|  | S8 | Pre | 0.23 | 0.24 | 0.13 | 2.54 |
|  |  | Post | 0.16 | 0.44 | 0.13 | 2.64 |
|  |  | Post/Pre | -0.13 | 0.53 | -0.08 | 3.42 |
| Fractional occupancy  (FO) | S1 | Pre | 0.06 | 0.76 | 0.02 | 3.99 |
|  |  | Post | 0.05 | 0.79 | 0.02 | 4.01 |
|  |  | Post/Pre | 0.12 | 0.56 | 0.07 | 3.61 |
|  | S2 | Pre | 0.03 | 0.87 | 0.04 | 3.88 |
|  |  | Post | -0.01 | 0.95 | -0.03 | 3.96 |
|  |  | Post/Pre | 0.02 | 0.92 | 0.02 | 4.01 |
|  | S3 | Pre | -0.18 | 0.37 | -0.12 | 2.84 |
|  |  | Post | 0.11 | 0.58 | 0.08 | 3.43 |
|  |  | Post/Pre | 0.54 | <0.01 | 0.32 | 0.31 |
|  | S4 | Pre | -0.39 | 0.05 | -0.27 | 0.59 |
|  |  | Post | -0.16 | 0.43 | -0.09 | 3.26 |
|  |  | Post/Pre | 0.33 | 0.09 | 0.22 | 1.16 |
|  | S5 | Pre | -0.09 | 0.64 | -0.09 | 3.35 |
|  |  | Post | 0.08 | 0.70 | 0.07 | 3.51 |
|  |  | Post/Pre | 0.14 | 0.49 | 0.12 | 2.74 |
|  | S6 | Pre | 0.11 | 0.59 | 0.05 | 3.83 |
|  |  | Post | -0.01 | 0.98 | -0.01 | 4.03 |
|  |  | Post/Pre | -0.06 | 0.78 | -0.04 | 3.88 |
|  | S7 | Pre | 0.07 | 0.74 | 0.05 | 3.78 |
|  |  | Post | 0.06 | 0.78 | 0.07 | 3.58 |
|  |  | Post/Pre | -0.01 | 0.96 | -0.01 | 4.03 |
|  | S8 | Pre | 0.07 | 0.74 | 0.05 | 3.83 |
|  |  | Post | -0.02 | 0.91 | -0.01 | 4.03 |
|  |  | Post/Pre | -0.24 | 0.23 | -0.17 | 1.94 |
| Mean interval length  (MIL) | S1 | Pre | -0.08 | 0.70 | -0.03 | 3.98 |
|  |  | Post | 0.00 | 1.00 | 0.03 | 3.92 |
|  |  | Post/Pre | -0.10 | 0.61 | -0.05 | 3.80 |
|  | S2 | Pre | 0.01 | 0.98 | -0.02 | 4.01 |
|  |  | Post | 0.01 | 0.95 | 0.02 | 3.99 |
|  |  | Post/Pre | -0.09 | 0.67 | -0.07 | 3.54 |
|  | S3 | Pre | 0.30 | 0.13 | 0.22 | 1.22 |
|  |  | Post | 0.09 | 0.65 | 0.07 | 3.62 |
|  |  | Post/Pre | -0.32 | 0.11 | -0.21 | 1.37 |
|  | S4 | Pre | 0.35 | 0.08 | 0.24 | 0.93 |
|  |  | Post | 0.19 | 0.34 | 0.13 | 2.54 |
|  |  | Post/Pre | -0.29 | 0.15 | -0.19 | 1.58 |
|  | S5 | Pre | 0.05 | 0.81 | 0.05 | 3.80 |
|  |  | Post | -0.10 | 0.63 | -0.09 | 3.30 |
|  |  | Post/Pre | -0.07 | 0.72 | -0.07 | 3.58 |
|  | S6 | Pre | -0.14 | 0.47 | -0.08 | 3.43 |
|  |  | Post | 0.03 | 0.93 | 0.01 | 4.03 |
|  |  | Post/Pre | -0.03 | 0.89 | -0.02 | 4.00 |
|  | S7 | Pre | -0.01 | 0.98 | -0.02 | 4.01 |
|  |  | Post | -0.08 | 0.68 | -0.08 | 3.43 |
|  |  | Post/Pre | 0.07 | 0.75 | 0.06 | 3.71 |
|  | S8 | Pre | -0.06 | 0.79 | -0.04 | 3.88 |
|  |  | Post | 0.07 | 0.74 | 0.04 | 3.88 |
|  |  | Post/Pre | 0.21 | 0.29 | 0.14 | 2.50 |
| Number of occurrences (NO) | S1 | Pre | 0.05 | 0.79 | 0.02 | 4.01 |
|  |  | Post | 0.05 | 0.83 | 0.01 | 4.04 |
|  |  | Post/Pre | 0.06 | 0.78 | 0.01 | 4.03 |
|  | S2 | Pre | 0.02 | 0.93 | 0.02 | 4.00 |
|  |  | Post | 0.02 | 0.94 | 0.00 | 4.04 |
|  |  | Post/Pre | 0.10 | 0.61 | 0.07 | 3.53 |
|  | S3 | Pre | -0.31 | 0.12 | -0.22 | 1.18 |
|  |  | Post | -0.14 | 0.50 | -0.10 | 3.16 |
|  |  | Post/Pre | 0.32 | 0.11 | 0.20 | 1.52 |
|  | S4 | Pre | -0.31 | 0.12 | -0.22 | 1.13 |
|  |  | Post | -0.18 | 0.37 | -0.13 | 2.69 |
|  |  | Post/Pre | 0.27 | 0.18 | 0.18 | 1.74 |
|  | S5 | Pre | -0.05 | 0.80 | -0.05 | 3.78 |
|  |  | Post | 0.03 | 0.89 | 0.05 | 3.80 |
|  |  | Post/Pre | -0.01 | 0.97 | 0.03 | 3.97 |
|  | S6 | Pre | 0.12 | 0.56 | 0.06 | 3.65 |
|  |  | Post | 0.08 | 0.70 | 0.07 | 3.58 |
|  |  | Post/Pre | -0.01 | 0.97 | -0.01 | 4.03 |
|  | S7 | Pre | 0.03 | 0.89 | 0.03 | 3.97 |
|  |  | Post | 0.05 | 0.80 | 0.04 | 3.85 |
|  |  | Post/Pre | 0.12 | 0.54 | 0.09 | 3.26 |
|  | S8 | Pre | 0.06 | 0.77 | 0.04 | 3.85 |
|  |  | Post | -0.07 | 0.72 | -0.04 | 3.88 |
|  |  | Post/Pre | -0.24 | 0.23 | -0.15 | 2.24 |

**Notes.** Correlations between Boost effect (BE) and HMM temporal parameters at Pre- and Post-learning sessions, and between BE and proportional changes in HMM parameter values from pre- to post-learning session (Post/Pre), for all States. Spearman’s Rho and p-value. Bayesian correlations (Kendall’s tau-b) and Bayes Factors BF01 are provided as indications of potential support for the null hypothesis (Supportive = BF > 3; Inconclusive = 0.33 < BF < 3; Against = BF < 0.33).


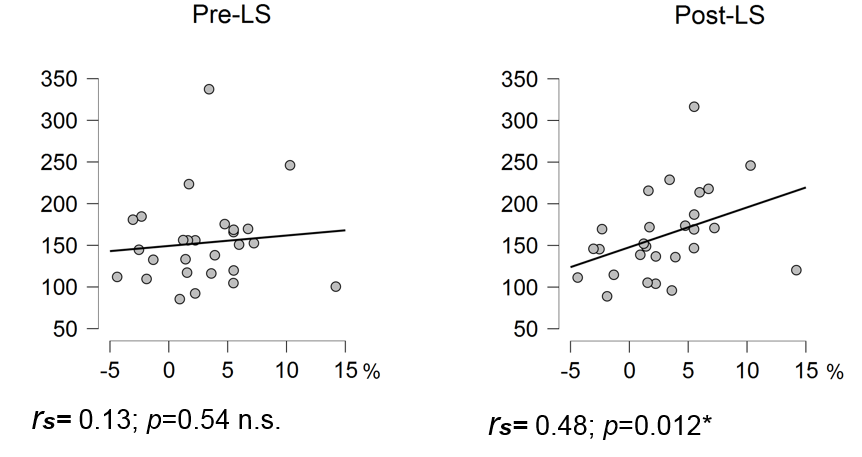


**Figure 6.** Correlations between the Boost effect (BE) and MLT for State 3. P-values were corrected *(*p < .013 (corrected for 4 HMM temporal parameters).* The figure was created using JASP version 0.14.1, JASP Team (2021), <https://jasp-stats.org>.


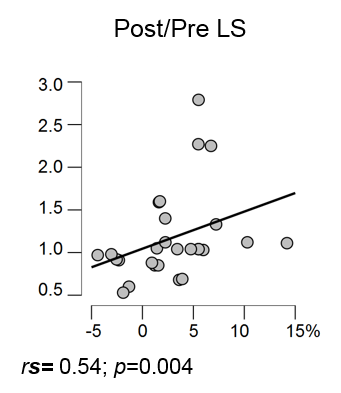


**Figure 7.** Correlations between the Boost effect (BE) and FO for State 3 proportional change (Post/pre). P-values were corrected *(*p < .013 (corrected for 4 HMM temporal parameters).* The figure was created using JASP version 0.14.1, JASP Team (2021), https://jasp-stats.org.

**VI. Baseline vs Best Motor Performance comparison**

In order to dissociate as much as possible motor sequential learning from mere motor execution, we reasoned that the first blocks in the learning session mostly reflect mere individual motor abilities since the sequence to be learned is not yet integrated at the procedural level, whereas with continued practice both motor and sequential components are at play to contribute to performance at the end of learning. Thus, we estimated a baseline (BL) performance as the average of FTT blocks 2 and 3 of the learning session (block 1 was not taken into account in this analysis as due to habituation to the task, variability was high with frequent stops and disruptions in the execution of the sequence), assuming that at that stage participants did not learn yet the sequence, which makes it a potential motor control situation. In the next step, we computed correlations between motor BL condition and state parameters measures, which essentially resulted in similar patterns than correlations with BMP (Table 6).

**Table 6. Correlations between HMM temporal parameters and behavioural indices of motor ability (BL) and/or motor and sequential learning (BMP)**

| **Measure** | **State** | **Session** | **Spearman’s rho**  **BMP** | **p values** | **Spearman’s rho**  **BL** | **p values** |
| --- | --- | --- | --- | --- | --- | --- |
| Mean life time  (MLT) | S1 | Pre | -0.59 | <0.01** | -0.40 | 0.04* |
|  |  | Post | -0.60 | <0.01** | -0.39 | 0.05* |
|  | S2 | Pre | 0.00 | 1.00 | 0.07 | 0.73 |
|  |  | Post | -0.02 | 0.94 | 0.05 | 0.80 |
|  | S3 | Pre | 0.36 | 0.07 | 0.40 | 0.04* |
|  |  | Post | 0.37 | 0.12 | 0.30 | 0.14 |
|  | S4 | Pre | -0.05 | 0.80 | -0.03 | 0.90 |
|  |  | Post | -0.11 | 0.56 | -0.05 | 0.78 |
|  | S5 | Pre | -0.03 | 0.88 | -0.08 | 0.70 |
|  |  | Post | -0.17 | 0.39 | -0.18 | 0.37 |
|  | S6 | Pre | 0.42 | 0.04* | 0.19 | 0.36 |
|  |  | Post | 0.60 | <0.01** | 0.45 | 0.02* |
|  | S7 | Pre | 0.50 | 0.01* | 0.46 | 0.02* |
|  |  | Post | 0.60 | <0.01** | 0.54 | <0.01* |
|  | S8 | Pre | 0.43 | 0.03* | 0.21 | 0.29 |
|  |  | Post | 0.42 | 0.03* | 0.25 | 0.21 |
| Fractional  occupancy  (FO) | S1 | Pre | -0.48 | 0.01* | -0.17 | 0.39 |
|  |  | Post | -0.51 | 0.01* | -0.18 | 0.38 |
|  | S2 | Pre | -0.03 | 0.89 | 0.15 | 0.46 |
|  |  | Post | 0.10 | 0.62 | 0.32 | 0.11 |
|  | S3 | Pre | 0.34 | 0.09 | 0.08 | 0.70 |
|  |  | Post | 0.36 | 0.06 | 0.18 | 0.37 |
|  | S4 | Pre | 0.01 | 0.95 | -0.08 | 0.70 |
|  |  | Post | -0.14 | 0.49 | -0.07 | 0.74 |
|  | S5 | Pre | -0.18 | 0.36 | -0.24 | 0.23 |
|  |  | Post | -0.23 | 0.25 | -0.17 | 0.40 |
|  | S6 | Pre | 0.39 | 0.05 | 0.06 | 0.77 |
|  |  | Post | 0.51 | 0.01* | 0.30 | 0.14 |
|  | S7 | Pre | 0.45 | 0.02* | 0.37 | 0.06 |
|  |  | Post | 0.48 | 0.01* | 0.42 | 0.03* |
|  | S8 | Pre | 0.42 | 0.03* | 0.11 | 0.57 |
|  |  | Post | 0.37 | 0.06 | 0.05 | 0.82 |
| Mean interval length  (MIL) | S1 | Pre | 0.47 | 0.01 * | 0.20 | 0.33 |
|  |  | Post | 0.58 | <0.01** | 0.29 | 0.14 |
|  | S2 | Pre | -0.01 | 0.97 | -0.16 | 0.41 |
|  |  | Post | -0.12 | 0.54 | -0.34 | 0.09 |
|  | S3 | Pre | -0.40 | 0.04* | -0.07 | 0.74 |
|  |  | Post | -0.38 | 0.05* | -0.20 | 0.31 |
|  | S4 | Pre | 0.04 | 0.83 | 0.18 | 0.37 |
|  |  | Post | 0.14 | 0.50 | 0.09 | 0.64 |
|  | S5 | Pre | 0.22 | 0.27 | 0.27 | 0.17 |
|  |  | Post | 0.26 | 0.20 | 0.22 | 0.27 |
|  | S6 | Pre | -0.32 | 0.10 | -0.01 | 0.95 |
|  |  | Post | -0.52 | 0.01 * | -0.33 | 0.10 |
|  | S7 | Pre | -0.48 | 0.01 * | -0.38 | 0.05* |
|  |  | Post | -0.39 | 0.05* | -0.38 | 0.05* |
|  | S8 | Pre | -0.41 | 0.03* | -0.12 | 0.55 |
|  |  | Post | -0.37 | 0.06 | -0.06 | 0.75 |
| Number of occurrence  (NO) | S1 | Pre | -0.45 | 0.02 | -0.14 | 0.48 |
|  |  | Post | -0.49 | 0.01* | -0.18 | 0.37 |
|  | S2 | Pre | -0.03 | 0.88 | 0.12 | 0.57 |
|  |  | Post | 0.11 | 0.57 | 0.30 | 0.13 |
|  | S3 | Pre | 0.40 | 0.04* | 0.10 | 0.65 |
|  |  | Post | 0.38 | 0.05* | 0.14 | 0.50 |
|  | S4 | Pre | -0.07 | 0.75 | -0.17 | 0.41 |
|  |  | Post | -0.15 | 0.45 | -0.12 | 0.56 |
|  | S5 | Pre | -0.23 | 0.25 | -0.24 | 0.23 |
|  |  | Post | -0.26 | 0.19 | -0.18 | 0.37 |
|  | S6 | Pre | 0.36 | 0.07 | 0.03 | 0.89 |
|  |  | Post | 0.58 | <0.01** | 0.38 | 0.05* |
|  | S7 | Pre | 0.33 | 0.09 | 0.30 | 0.13 |
|  |  | Post | 0.36 | 0.06 | 0.37 | 0.06 |
|  | S8 | Pre | 0.40 | 0.04 | 0.12 | 0.56 |
|  |  | Post | 0.36 | 0.06 | 0.06 | 0.76 |

**Notes.** Correlations between HMM temporal parameters and behavioural indices of motor ability (BL) / motor and sequential learning (BMP) at Pre- and Post-learning sessions for 8 States. Spearman’s Rho and *p*-values (**p - not corrected; **p - corrected by factor 32).*

**VII. State transitions**

We also examined whether motor learning is reflected in changes to the pattern of transitions between the states, i.e., we computed a transition probability matrix ^4^ and compared the transitions before and after learning (Figure 8). No result passed the statistical threshold (corrected for 8 states x 7 probabilities).


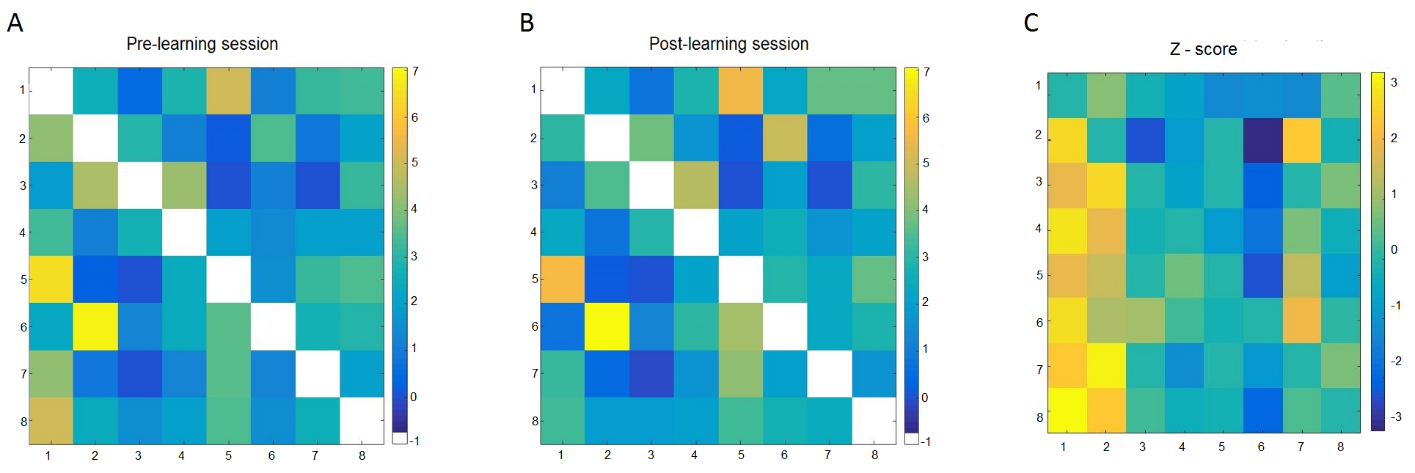


**Figure 8.** State transition matrices of the 8 HMM states for Pre- **(A)** and Post-learning **(B)** resting state sessions generated using MATLAB R2016a, The MathWorks, https://www.mathworks.com/. The matrix displays the probabilities (% on the scale) of transitioning to any distinct state given the current state. White squares represent the probability of remaining in the same state. **(C)** Z -scores of the contrast between pre- and post-learning sessions.

**References**

1. Cleeremans, A. & Mcclelland, J. L. Learning the structure of event sequences. *J. Exp. Psychol.* **120**, 235–253 (1991).

2. Sakai, K., Kitaguchi, K. & Hikosaka, O. Chunking during human visuomotor sequence learning. *Exp Brain Res* **152**, 229–242 (2003).

3. Gobet, F. *et al.* Chunking mechanisms in human learning. *Trends Cogn. Sci.* **5**, 236–243 (2001).

4. Baker, A. P. *et al.* Fast transient networks in spontaneous human brain activity. *Elife* **3**, e01867 (2014).
